# Fresh and cryopreserved ovarian tissue transplantation for preserving reproductive and endocrine function: a systematic review and individual patient data meta-analysis

**DOI:** 10.1093/humupd/dmac003

**Published:** 2022-02-24

**Authors:** Hajra Khattak, Rosamund Malhas, Laurentiu Craciunas, Yousri Afifi, Christiani A Amorim, Simon Fishel, Sherman Silber, Debra Gook, Isabelle Demeestere, Olga Bystrova, Alla Lisyanskaya, Georgy Manikhas, Laura Lotz, Ralf Dittrich, Lotte Berdiin Colmorn, Kirsten Tryde Macklon, Ina Marie Dueholm Hjorth, Stine Gry Kristensen, Ioannis Gallos, Arri Coomarasamy

**Affiliations:** 1 Tommy’s National Centre for Miscarriage Research, Institute of Metabolism and Systems Research, University of Birmingham, Birmingham, UK; 2 Birmingham Women’s and Children’s NHS Foundation Trust, Birmingham, UK; 3 Population Health Sciences Institute, Newcastle University, Newcastle upon Tyne, UK; 4 Pôle de Recherche en Gynécologie, Institut de Recherche Expérimentale et Clinique, Université Catholique de Louvain, Brussels, Belgium; 5 CARE Fertility Group, Nottingham, UK; 6 School of Pharmacy and Biomolecular Sciences, Liverpool John Moores University, Liverpool, UK; 7 Infertility Centre of St. Louis, Saint Louis, MO, USA; 8 Reproductive Services/Melbourne IVF, The Royal Women’s Hospital, Parkville, VIC, Australia; 9 Research Laboratory on Human Reproduction, Faculty of Medicine, Université Libre de Bruxelles (ULB), Brussels, Belgium; 10 AVA-PETER Fertility Clinic, Saint-Petersburg, Russia; 11 Division of Gynecologic Oncology, Saint-Petersburg City Oncology Clinic, Saint-Petersburg, Russia; 12 Department of Oncology of the First Pavlov State Medical University of Saint-Petersburg, Saint-Petersburg, Russia; 13 Department of Obstetrics and Gynecology, Erlangen University Hospital, Friedrich Alexander University of Erlangen-Nuremberg, Erlangen, Germany; 14 The Fertility Clinic, University Hospital of Copenhagen, Rigshospitalet, Copenhagen, Denmark; 15 Department of Obstetrics and Gynaecology, Aarhus University Hospital, Aarhus, Denmark; 16 Laboratory of Reproductive Biology, The Juliane Marie Centre for Women, Children and Reproduction, University Hospital of Copenhagen, Rigshospitalet, Copenhagen, Denmark

**Keywords:** ovarian tissue, cryopreservation, transplantation, premature ovarian insufficiency, fertility preservation, menopause, oncofertility

## Abstract

**BACKGROUND:**

Ovarian tissue cryopreservation involves freezing and storing of surgically retrieved ovarian tissue in liquid or vapour nitrogen below –190°C. The tissue can be thawed and transplanted back with the aim of restoring fertility or ovarian endocrine function. The techniques for human ovarian tissue freezing and transplantation have evolved over the last 20 years, particularly in the context of fertility preservation in pre-pubertal cancer patients. Fresh ovarian tissue transplantation, using an autograft or donor tissue, is a more recent development; it has the potential to preserve fertility and hormonal function in women who have their ovaries removed for benign gynaecological conditions. The techniques of ovarian tissue cryopreservation and transplantation have progressed rapidly since inception; however, the evidence on the success of this intervention is largely based on case reports and case series.

**OBJECTIVE AND RATIONALE:**

The aim of this study was to systematically review the current evidence by incorporating study-level and individual patient-level meta-analyses of women who received ovarian transplants, including frozen–thawed transplant, fresh or donor graft.

**SEARCH METHODS:**

The review protocol was registered with PROSPERO (CRD42018115233). A comprehensive literature search was performed using MEDLINE, EMBASE, CINAHL and Cochrane Central Register of Controlled Trials from database inception to October 2020. Authors were also contacted for individual patient data if relevant outcomes were not reported in the published manuscripts. Meta-analysis was performed using inverse-variance weighting to calculate summary estimates using a fixed-effects model.

**OUTCOMES:**

The review included 87 studies (735 women). Twenty studies reported on ≥5 cases of ovarian transplants and were included in the meta-analysis (568 women). Fertility outcomes included pregnancy, live birth and miscarriage rates, and endocrine outcomes included oestrogen, FSH and LH levels. The pooled rates were 37% (95% CI: 32–43%) for pregnancy, 28% (95% CI: 24–34%) for live birth and 37% (95% CI: 30–46%) for miscarriage following frozen ovarian tissue transplantation. Pooled mean for pre-transplant oestrogen was 101.6 pmol/l (95% CI: 47.9–155.3), which increased post-transplant to 522.4 pmol/l (95% CI: 315.4–729; mean difference: 228.24; 95% CI: 180.5–276). Pooled mean of pre-transplant FSH was 66.4 IU/l (95% CI: 52.8–84), which decreased post-transplant to 14.1 IU/l (95% CI: 10.9–17.3; mean difference 61.8; 95% CI: 57–66.6). The median time to return of FSH to a value <25 IU/l was 19 weeks (interquartile range: 15–26 weeks; range: 0.4–208 weeks). The median duration of graft function was 2.5 years (interquartile range: 1.4–3.4 years; range: 0.7–5 years). The analysis demonstrated that ovarian tissue cryopreservation and transplantation could restore reproductive and hormonal functions in women. Further studies with larger samples of well-characterized populations are required to define the optimal retrieval, cryopreservation and transplantation processes.

**WIDER IMPLICATIONS:**

Ovarian tissue cryopreservation and transplantation may not only be effective in restoring fertility but also the return of reproductive endocrine function. Although this technology was developed as a fertility preservation option, it may have the scope to be considered for endocrine function preservation.

## Introduction

There is an increase in the number of young girls and women diagnosed with cancer globally. In 2020 alone, ∼0.9 million cases of new cancers were diagnosed worldwide in women aged 0–39 years (crude and age-standardized incidence rate per 100 000) (International Agency for Research on Cancer (IARC) and World Health Organization (WHO), 2021). Ground-breaking research into anti-cancer therapies has resulted in a significant increase in survival rates of young female cancer participants, which has brought into focus the need for maintaining quality of life in these women (Stam *et al.*, 2001; Langeveld *et al.*, 2002; Nieman *et al.*, 2007). Unfortunately, gonadotoxic anti-cancer therapies pose substantial risk to the fertility prospects of girls and young women. Oocyte cryopreservation can be offered to post-pubertal women; however, this is not an option for pre-pubertal girls. Over the last two decades, a new method of fertility preservation has been developed to cryopreserve ovarian cortical tissue, and this option has the advantage of being suitable for pre-pubertal girls. The surgically retrieved ovarian tissue is prepared by separating the cortex from the medulla. The ovarian cortex, which contains thousands of primordial follicles in girls and young women, is then cut into strips, dehydrated in cryoprotectant solution and cryopreserved (frozen) using controlled rate freezing (slow) or vitrification (ultrarapid). The cryopreserved tissue is stored in vials and once the cancer treatment is concluded and the patient is deemed disease-free by their oncologist, the tissue can be thawed and transplanted back to restore fertility and endocrine function. Transplantation process may involve surgically transplanting ovarian tissue onto the remaining ovary (orthotopic), pelvic side wall, subcutaneously or intramuscularly (heterotopic). To date, thousands of girls and young women have had their ovarian tissue cryopreserved (Gellert *et al.*, 2018; Andersen *et al.*, 2019) and for those not given the option, there is a significant level of regret (Jayasuriya *et al.*, 2019). Ovarian tissue cryopreservation and transplantation have shown promise in preserving fertility and restoring endocrine function (Donnez *et al.*, 2004; Meirow *et al.*, 2005; Silber *et al.*, 2005; Oktay *et al.*, 2011; Andersen *et al.*, 2012; Silber, 2012; Silber *et al.*, 2015). The frozen–thawed ovarian tissue grafts are capable of endocrine function, producing viable oocytes for up to 7 years or even longer (Andersen *et al.*, 2012; Grynberg *et al.*, 2012; Donnez and Dolmans, 2015; Jensen *et al.*, 2015). This procedure has therefore enabled women to have their biological children, while restoring physiological ovarian hormonal function.

Fresh ovarian transplants have also been used to establish endocrine function and fertility in recipients with premature ovarian insufficiency (POI). The first successful fresh human ovary transplantation was reported in 2005 in monozygotic twins (Silber *et al.*, 2005). The same centre performed further fresh transplants in eight participants resulting in 11 healthy babies. In recent years, multiple centres have reported a series of fresh ovarian transplants with evidence of endogenous hormone production (Callejo *et al.*, 2001; Donnez *et al.*, 2005; Mhatre and Mhatre, 2006; Sánchez *et al.*, 2007; Silber *et al.*, 2008; Donnez *et al.*, 2011a; Andersen *et al.*, 2012; Almodin *et al.*, 2015; Silber *et al.*, 2010).

Despite ovarian tissue cryopreservation and transplantation being available for two decades, there is a marked variation in the delivery of this procedure worldwide. Most of the data are based on case reports from specialized centres with expertise in providing this procedure, but there are many unpublished cases. The objective of this review was to synthesize the existing evidence on the use of fresh and cryopreserved ovarian tissue transplantation, using study-level data and individual patient data (IPD).

## Methods

### PROSPERO registration and systematic search

The review protocol was registered with PROPERO (CRD42018115233) on 15 November 2018 (Khattak *et al.*, 2018). A comprehensive literature search was performed using MEDLINE, EMBASE, CINAHL and Cochrane Central Register of Controlled Trials from database inception to October 2020.

The databases were searched using the following key words and medical subject heading (MeSH) terms: ovarian, cryopreservation, transplantation, fresh transplantation, pregnancy, live birth and ovarian function. The search strategy for MEDLINE is available as Supplementary Table SI. The search was completed by screening the reference lists of all relevant publications.

### Inclusion and exclusion criteria

Inclusion and exclusion criteria for studies were established before the literature search was conducted. Study selection was carried out by two independent reviewers (H.K. and R.M.). Any disagreements about inclusion were resolved by consensus or arbitration by a third reviewer (L.C.).

All studies that reported fertility or endocrine outcomes from either fresh or frozen–thawed ovarian transplants for at least one participant were included. These comprised cohort studies, observational studies, case reports, case series, conference abstracts and grey literature (irrespective of country of origin, affiliations of authors, language or year of publication). Commentaries, editorials, correspondence and letters were excluded. When more than one publication originated from the same centre, population or cohort, reports were individually assessed to identify and remove duplicates. This was also double-checked by cross referencing and contacting the authors directly for clarification, and with the most recent or complete publication being selected.

### Primary and secondary outcomes

For each patient identified and included in the study, the aim was to collect data on ovarian reproductive function, such as pregnancy, live births and miscarriages, and endocrine function, such as oestrogen, progesterone, FSH, LH and anti-Müllerian hormone (AMH) levels. The return of hormonal function was defined by an increase in oestrogen, and a decrease in FSH and LH along with return of menstruation. Although the accuracy of values of FSH has not been assessed robustly in the literature, for the purpose of this review, the ESHRE guidelines on management of women with POI is used as a reference (European society of human reproduction and embryology (ESHRE) Guideline Group on POI *et al.*, 2016). A return of hormonal activity was described as women having achieved an FSH of <25 international units per litre (IU/l) post-transplant, LH of <15 IU/l and oestrogen of >200 picomoles per litre (pmol/l). Furthermore, characteristics of participants that could have potential modifier effects on return of reproductive and ovarian endocrine function were pre-specified. These included variables such as age at cryopreservation, age at transplantation, cryopreservation before gonadotoxic chemotherapy, amount of ovary transplanted and site of transplant. Studies that only reported an aggregate for the cohorts were grouped separately.

### Data extraction and quality assessment

Data extraction was performed in duplicate using a pre-defined piloted proforma. Both H.K. and R.M. extracted the data. Modified Newcastle–Ottawa scale was used for assessing the quality of the studies (Wells *et al.*).

### IPD requests

The authors of case reports and cohort studies were contacted and requested to provide IPD if the relevant outcomes were not available in the publication. If we were unsuccessful in acquiring IPD, the studies were included in the aggregate data meta-analysis. Patient-level data were requested from 16 centres. The data were provided for 220 women from six centres (USA, Belgium, Australia, Russia, Germany and Denmark).

### Data analysis and presentation

Mean and SD, or median, interquartile range (IQR) and range were used to summarize the data from studies that reported ≥5 cases. All the outcomes were converted to a standard unit for analysis; for FSH and LH, IU/l and for oestrogen, pmol/l. The pooled outcomes were calculated as mean difference (MD) for FSH, LH and oestrogen using the inverse variance method with 95% CIs and a fixed effects model (Demets, 1987). Risk Ratios (RRs) with 95% CIs were calculated for pregnancy rates in relation to age (≤35 years and >35 years) at retrieval, reported as dichotomous variables with the inverse variance method under the fixed-effects model. Review Manager 5.3 was used for calculating MDs and RRs (The Cochrane Collaboration, 2014).

To explore heterogeneity, χ^2^ test was used and significance was set at *P* < 0.05, where *I*^2^ was used for quantifying heterogeneity (Higgins and Thompson, 2002). Meta-analysis for pooled estimates for overall pregnancy rates, live birth rates and miscarriages were calculated using inverse-variance weighting to calculate the fixed-effects summary estimates. Statistical analyses were performed using Stata statistical package (Version 17, StataCorp, College Station, TX, USA) and Review Manager (Revman) software (The Cochrane Collaboration, 2014).

## Results

**Figure 1.**
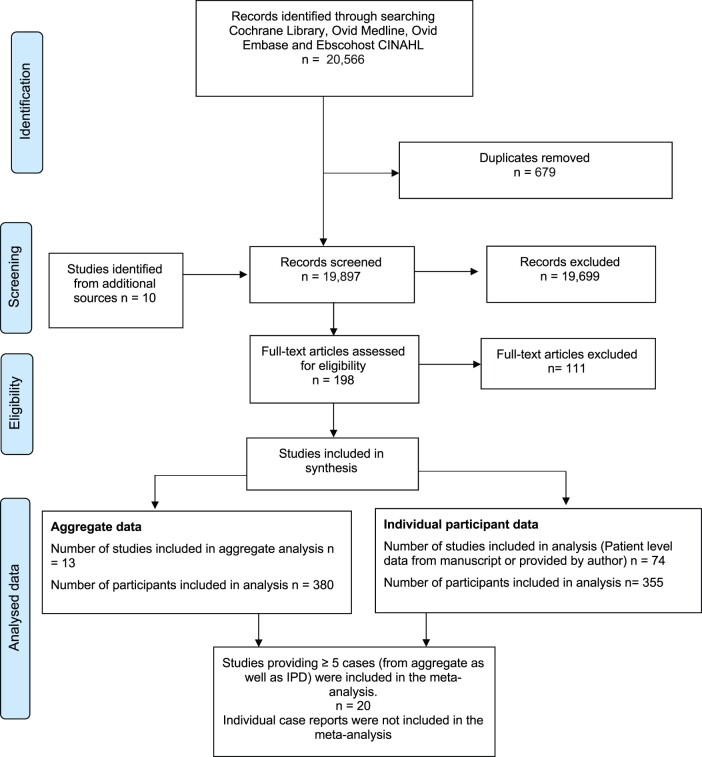
PRISMA flow diagram of search and selection strategy.

A total of 20 566 records were identified through the literature search. After removing 679 duplicates and addition of 10 studies from sources outside the search, 19 897 titles and abstracts were screened. After excluding studies that were not relevant, 198 full text articles were assessed for eligibility. Out of these, 87 studies (735 women) were included in the review. We were able to extract IPD for 355 women and study-level data for 380 women. Studies that reported ≥5 cases of ovarian transplants were included in the statistical analysis (568 women). The characteristics of studies included in the meta-analysis are reported as Supplementary Table SII. The PRISMA flow chart for the study is presented in Fig. 1.

### Reproductive outcomes after ovarian tissue transplantation

#### Pregnancies

**Figure 2.**
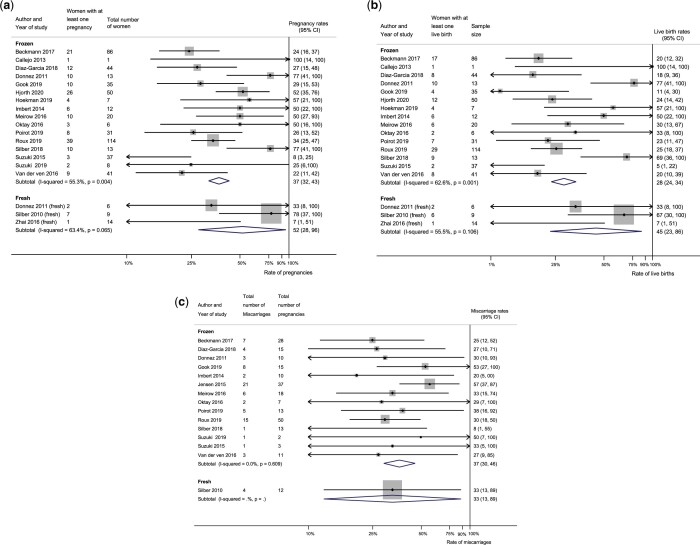
**Meta-analysis of reproductive outcomes from fresh and frozen human ovarian transplants.** (**a**) Pregnancy rates, (**b**) live birth rates and (**c**) miscarriage rates.

Eighteen studies (547 women) were included in meta-analysis for reproductive outcomes and at least one pregnancy was reported in 184 women (Fig. 2a). The pregnancy rate for frozen transplants was 37% (95% CI: 32–43%) and for fresh transplants was 52% (95% CI: 28–96%). Some women achieved more than one pregnancy giving an overall of 290 pregnancies reported in the literature.

#### Live births

Seventeen studies (539 women) were included in the meta-analysis and at least one live birth was reported in 134 women (Fig. 2b). The live birth rate for frozen transplants was 28% (95% CI: 24–34%) and for fresh transplants was 45% (95% CI: 23–86%). Some women achieved more than one live birth giving a total of 166 live births from women included in meta-analysis. The median number of live births per patient from frozen–thawed transplant was 1 (range: 1–4) and the median number of live births per patient from fresh transplant was also 1 (range: 1–3). Apart from the 17 studies, we also found case reports that described a further 34 live births. Overall, 189 live births have been reported in the literature.

#### Miscarriages

Fifteen studies reported miscarriage rates. The mean age at cryopreservation in women who had miscarriages was 27.8 years (SD: 5.8). Miscarriage rate for frozen transplants was 37% (95% CI: 30–46%) and for fresh transplants was 33% (95% CI: 13–89%) as presented in Fig. 2c.

### Endocrine function after ovarian tissue transplantation

#### Oestrogen

**Figure 3.**
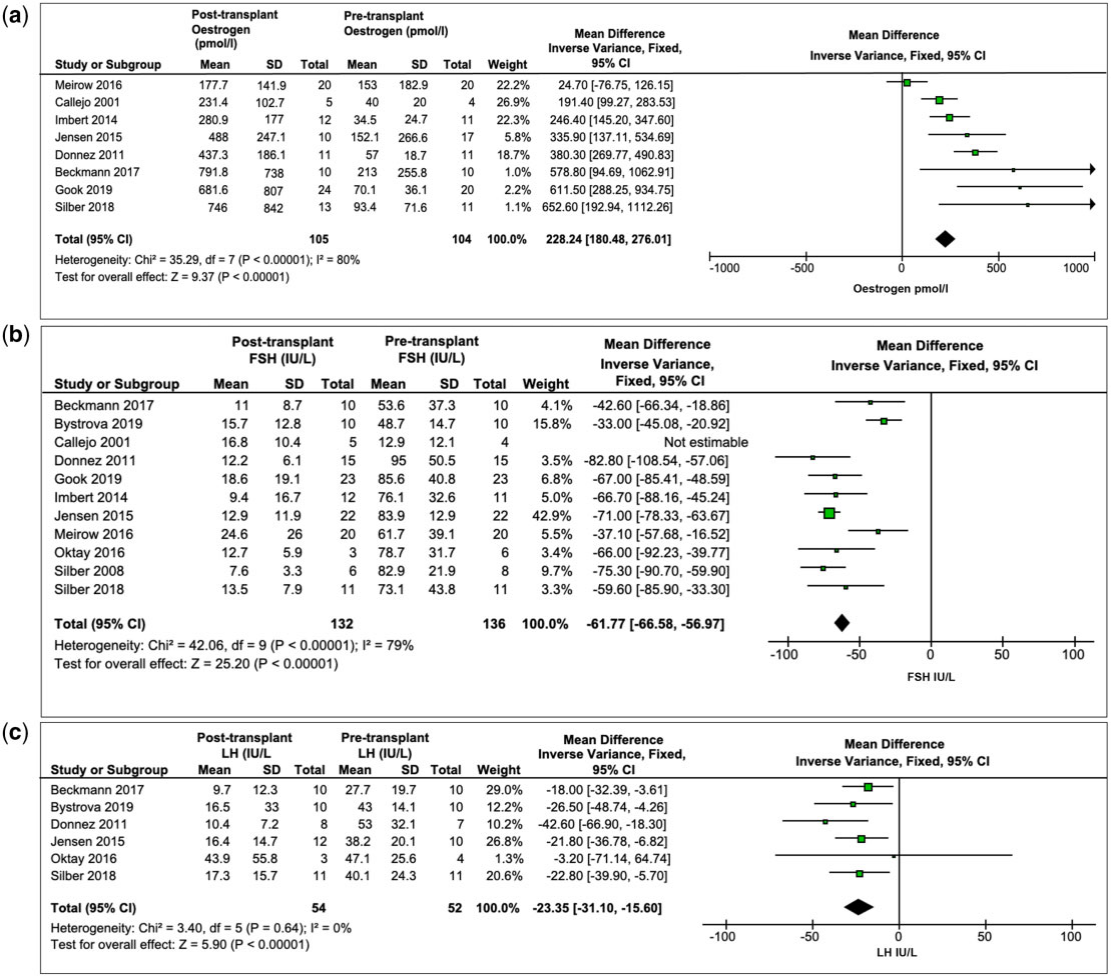
**Evidence of return of hormonal function after human ovarian transplantation.** (**a**) an increase in oestrogen (pmol/l) post-transplant, (**b**) a decrease in FSH (IU/l) post-transplant and (**c**) a decrease in LH (IU/l) post-transplant.

Eight studies (Fig. 3a) reported the levels of oestrogen pre-transplantation (104 women) and post-transplantation (105 women). Pooled mean for pre-transplant oestrogen was 101.6 pmol/l (95% CI: 47.9–155.3), which increased post-transplant to 522.4 pmol/l (95% CI: 315.4–729; MD: 228.24; 95% CI: 180.5–276). An increase in oestrogen of >200 pmol/l was noted in 117 women (75%) post graft. The median time to return of oestrogen to a value >200 pmol/l was 19.5 weeks (IQR: 14–24 weeks; range: 5–208 weeks).

#### FSH

Eleven studies (Fig. 3b) reported FSH pre-transplantation (136 women) and post-transplantation (132 women). Pooled means of pre-transplant FSH was 68.4 IU/l (95% CI: 52.8–84), which decreased post-transplant to 14.1 IU/l (95% CI: 10.9–17.3; MD: 61.8; 95% CI: 57–66.6) with substantial heterogeneity, *I*^2^ = 79% (*P* = 0.0001). Overall FSH levels post-transplant were reported for 187 out of 735 women. A decrease in FSH below 25 IU/l was achieved in 72% (135/187 women). The median time to return of FSH to a value <25 IU/l was 19 weeks (IQR: 15–26 weeks; range 0.4–208 weeks).

#### LH

Six studies (Fig. 3c) reported LH pre-transplantation (52 women) and post-transplantation (54 women). Pooled mean for pre-transplant LH was 41.5 IU/l (95% CI: 32.5–50.5), which decreased post-transplant to 19 IU/l (95% CI: 5.8–32.2; MD: 23.4; 95% CI: 15.6–31.1), heterogeneity *I*^2^ = 0% (*P* = 0.64). Overall, LH values post-transplantation were described in 69 out of 735 women. A decrease in LH below 15 IU/l was achieved in 46 out of 69 women (67%). The median time to return of LH to a value <15 IU/l was 19.5 weeks (IQR: 14–27 weeks; range: 8–156 weeks).

Additionally, we were able to collate reproductive outcomes based on whether participants achieved an FSH of ≤25 IU/l and compared to those who had FSH of >25 IU/l post-transplantation. It was found that an FSH of ≤25 IU/l was reported in 128 women, 64 (50%) of whom were able to achieve at least one pregnancy and 44 (34%) at least one live birth (Supplementary Table SIII).

For reproductive outcomes in relation to levels of oestrogen, we only assessed the data of participants who were truly menopausal before transplantation (levels of oestrogen < 100 pmol/l) (Middle and Kane 2009). Data post-transplant were then divided into two categories: participants who achieved oestrogen level of ≥200 pmol/l post-transplantation and those who did not. It was found that 56 participants achieved an oestrogen of ≥200 pmol/l post-transplantation, 19 (34%) of whom achieved at least one pregnancy and 15 (27%) at least one live birth (Supplementary Table SIV).

#### AMH

Only one study provided enough data for AMH pre- and post-transplantation (Beckmann *et al.*, 2017b). We found that even in those women who had an AMH of < 1 ng/ml pre-transplant, 19 pregnancies were observed in 71 patients (pregnancy rate = 27%).

#### Return of menstruation

Menstrual activity was reported as an outcome in 273 out of 735 women. It was noted that 196 out of 273 (72%) women were reported to have resumed menstruation. The median time to return of menstrual activity was 18 weeks (IQR: 14–22 weeks; range: 3–48 weeks). The return of menstrual activity coincides with that of hormonal function (median time to return of FSH to a value of <25 IU/l is 19 weeks and LH < 15 IU/l was 19.5 weeks).

### How fresh and frozen–thawed transplants differ in restoring hormonal and fertility outcomes

Through the literature search, we identified 45 fresh transplants, 11 of which used a graft from a donor (twin sister). Fifteen pregnancies and eight live births were reported for participants receiving fresh ovarian transplantation. Two studies that included five or more participants receiving fresh transplants were included in meta-analysis (Supplementary Figs S1 and S2). Pooled mean for oestrogen before transplantation was 54.8 pmol/l (SD: 7.6) and after fresh ovarian transplant, 403.3 pmol/l (SD: 128.3), (MD: 307.31; 95% CI: 159.78–454.85; *z* = 4.08; *I*^2^ = 0%), as shown in Supplementary Fig. S1. Three studies reported FSH in women having received fresh transplants but only two studies described outcomes in women who were menopausal (FSH > 25 IU/l) at the time of transplantation (Supplementary Fig. S2). Pooled mean for FSH in these women pre-transplant was 83.9 IU/l (SD 2.9) and post-transplant 9.1 IU/l (SD 2.1) (MD: 74.65; 95% CI: –49.91 to 99.39; *z* = 5.91; *I*^2^ 0%).

### Duration of graft function

Duration of the ovarian graft function was reported in 19 studies (181 women). In 15 studies, the authors reported the exact duration (or median duration if more than one patient was reported). The median duration of function was 2.5 years (IQR: 1.4–3.4 years), range: 0.7–5 years. The mean age at cryopreservation for this group of women was 27.1 years (SD: 6.8). A further three studies, including 26 women with a mean age at cryopreservation 30.3 years (SD: 2.5), reported a range with pooled duration of function being 1.2–7.7 years. A case series of three participants who received fresh ovarian transplants (patient’s own ovary) between the ages of 1–5 years experienced menarche as well as 13–15 years of duration of function (Laufer *et al.*, 2010).

### Outcomes based on factors that affect fertility and likelihood of return of endocrine function

#### Age at ovarian tissue retrieval for cryopreservation

**Figure 4.**
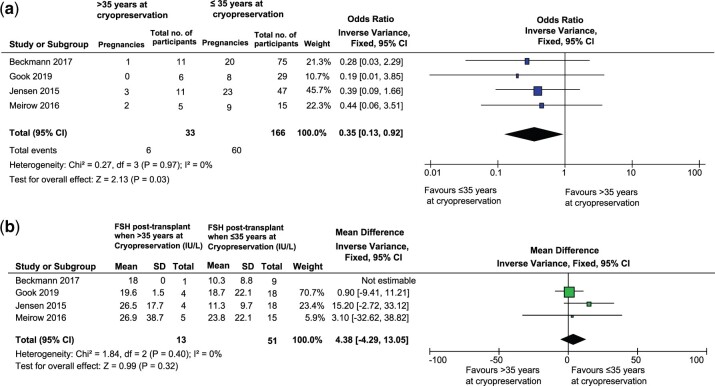
**Comparisons of outcomes in participants who had ovarian tissue cryopreserved before the age of 35 years, to those who had ovarian tissue cryopreservation after 35 years of age.** (**a**) Comparison of pregnancy rates and (**b**) Comparison of return of hormonal function (FSH).

It was found that out of 735 women included in the review, age was provided for 319 women at participant level data. Of these, 283 had their ovarian tissue retrieved for cryopreservation at ≤35 years of age. A subgroup of four studies that reported data on participants age at cryopreservation and transplantation (Fig. 4a and b) were included in meta-analysis. We found that pregnancy rates were higher in participants in whom ovarian tissue was cryopreserved at ≤35 years of age, with results being statistically significant (Odds Ratio: 0.35; 95% CI: 0.13–0.92; *z* = 2.13; *P* = 0.03, *I*^2^ = 0%). Return of hormonal function is shown as a decrease in FSH and was lower in the group that had ovarian tissue frozen at ≤35 years of age (MD: 4.38; 95% CI: –4.29 to 13.05; *z* = 0.99; *P* = 0.32, *I*^2^ = 0%) (Fig. 4b).

#### Mode of conception

Mode of conception was provided clearly in 276 pregnancies. It was noted that 199 (69%) pregnancies were conceived naturally, whereas ART was used for 90 pregnancies (Supplementary Table SV).

#### Anti-cancer therapy before retrieval

Whether a participant received anti-cancer therapy before retrieval of ovarian tissue was reported in 122 out of 735 participants. It was found that 56 out of 122 patients (46%) had received anti-cancer treatment before ovarian tissue cryopreservation. Thirty-five pregnancies and 24 live births were reported in these women. In women with live births, 11 had Hodgkin’s lymphoma, 5 had non-Hodgkin’s lymphoma, 1 had microscopic polyangiitis, 1 had acute myeloid leukaemia (AML) and 1 had Wilms’ tumour. Mean age at cryopreservation for women who received chemotherapy before tissue freezing and achieved live birth was 29 years (SD: 6). We were able to perform meta-analysis on data from five studies that reported reproductive and endocrine outcomes based on whether the women received anti-cancer treatment before cryopreservation or not. Although the results were not statistically significant, a decrease in FSH, an increase in oestrogen and increased pregnancy rates were noted in participants who did not receive anti-cancer therapy before cryopreservation (Supplementary Figs S3, S4, and S5).

#### Type of cancer

The five most common cancers at participant level included breast cancer, cervical cancer, non-Hodgkin’s lymphoma, ovarian cancer and sickle cell anaemia. There was insufficient IPD across the cohorts to be able to perform a meta-analysis and provide pregnancies and live birth rates based on type of cancer. The sums of pregnancies and live birth rates in women with the five most common cancers based on IPD are described in Supplementary Table SVI. Of all the cancer cases reported, we found no live births in women who had suffered with cervical cancer. Given that cervical cancer affects women of reproductive age, transplanting cryopreserved ovarian tissue may help with symptoms of early menopause. To assess endocrine function, we conducted meta-analysis on two studies that included more than five participants that had cervical cancer (Supplementary Fig. S6). There was a significant reduction in FSH levels; pooled mean for pre-transplant FSH was 69.1 IU/l (95% CI: 44.5–93.5), which decreased to 17.6 IU/l (95% CI: 11.7–32.6; MD: 37.1; 95% CI: 49.8–24.3). The return of hormonal function in this cohort is therefore promising and ovarian tissue cryopreservation and transplantation may be beneficial to these women to help with their menopausal symptoms.

#### Amount of ovary transplanted to achieve reproductive or endocrine function

There was inconsistency in how the amount of ovarian tissue transplanted was measured. The authors described the amount of the ovarian tissue in volume, strips, fragments, pieces, biopsies or sections. Not all authors reported a 3D measurement of the ovarian graft. Owing to the variation in size of the ovary and follicular count at baseline, it is not possible to estimate the optimal amount of tissue for achieving desirable reproductive and hormonal outcomes.

#### Slow freezing versus vitrification

In our review we found 13 cases in whom vitrification was used to cryopreserve ovarian tissue (Kiseleva *et al.*, 2015; Silber *et al.*, 2018; Iwahata *et al.*, 2020). Five pregnancies and two live births were reported. Based on the data available, meta-analysis showed that the cumulative pregnancy rate for ovarian tissue cryopreserved using slow freezing was 37% as compared to vitrification, which was 44% (Supplementary Figs S7 and S8).

### Surgical approaches for ovarian tissue retrieval and transplantation

#### Surgical technique

The surgical approach for retrieval of ovarian tissue was reported for 237 out of 735 women, with 225 women (95% having had laparoscopy and 12 (5%) having had laparotomy). As for transplantation, surgical approach was reported for 323 women; 205 (64%) had laparoscopy and 95 (29%) had laparotomy. Other sites and techniques are listed in Supplementary Table SVII. The site of transplant was explicitly reported in 440 participants and described as transplant onto a remaining ovary (orthotopic), pelvic side wall or peritoneal pocket (heterotopic), transplant on two sites (orthotopic + heterotopic), transplant at three sites (remaining ovary, pelvic side wall and abdominal wall), subcutaneous, subdermal and intramuscular (rectus abdominis and deltoid muscles).

#### Transplant on to remaining ovary

Of the 440 women, 175 had ovarian tissue transplanted onto their remaining postmenopausal ovary. FSH levels pre- and post-transplantation were reported in 55 participants, with mean FSH of 71.5 IU/l (SD 44.6) pre-transplant and 25.3 (SD: 28.5) post-transplantation. Oestrogen at pre-transplantation and post-transplant was reported in 33 of the 175 participants. Mean oestrogen before transplant was 104.9 pmol/l (SD: 143.7) and post-transplant 387.5 pmol/l (SD: 419.6). Fifty-two pregnancies and 34 live births have been recorded from transplant onto the remaining ovary.

#### Pelvic side wall or peritoneal pocket

Ovarian tissue was transplanted onto the pelvic side wall or peritoneal pockets in 184 women. Oestrogen levels at baseline and post-transplant were described in 22 participants. The mean oestrogen level before transplant was 207.5 pmol/l (SD: 245.1) and post-transplant 1204.4 pmol/l (SD: 1164.3). FSH was reported in 25 participants, with mean pre-transplant FSH of 58.8 IU/l (SD: 38.5) and post-transplant 14.4 IU/l (SD: 13.2). Forty-one pregnancies and 37 live births were reported.

#### Transplant onto two sites: pelvic side wall and remaining ovary

Fifty participants had their ovarian tissue transplanted in the pelvic peritoneum as well as the remaining ovary. The mean oestrogen level pre-transplantation in these participants was 172 pmol/l (SD: 144) and post-transplant 1922 pmol/l (SD: 3257.9), reported in 14 participants. FSH was reported in 24 participants with mean pre-transplant 84.7 IU/l (SD: 47.6) and post-transplant 20.5 IU/l (SD: 21.1). Twenty-eight pregnancies and 10 live births were reported.

#### Transplant onto three sites: pelvic side wall, remaining ovary and abdominal wall

Eight participants received ovarian transplantation in three sites during one operation. FSH was reported in seven participants with a mean of 62.9 IU/l (SD: 28.1) pre-transplant and 10.8 IU/l (SD 8.5) post-transplant. There was insufficient data to report on oestrogen post-transplantation. Four pregnancies and one live birth were reported. The rest of the transplant sites (abdominal, subcutaneous and intramuscular) are described in the Supplementary Table SVIII.

### Risks of surgery

The included studies did not report any specific complications related to ovarian transplantation other than those of gynaecological laparotomy and laparoscopic surgery. The only complications reported thus far were those of skin infection and injury to surrounding organs (Rosendahl *et al.*, 2011; Dolmans *et al.*, 2013; Hoekman *et al.*, 2020). The largest dataset from ‘FertiProtekt’ network (a collaborative network of German speaking countries) that included 71 transplantations in 58 participants did not highlight any ovarian transplantation-related complications (Beckmann *et al.*, 2017b).

### Risk of subsequent cancers in the ovarian graft

Through our literature search, we found two cases of cancers reported in the transplanted ovarian graft. A case report diagnosed the recurrence of granulosa cell tumour in a patient at caesarean section delivery. The patient had not received any adjuvant chemotherapy before oophorectomy for ovarian tissue cryopreservation (Stern *et al.*, 2014). In another case, a patient who was treated for Ewing’s sarcoma and had ovarian tissue cryopreserved before receiving chemotherapy, presented with an ovarian mucinous cystadenoma in the transplant (Fajau-Prevot *et al.*, 2017).

### Worldwide activity

**Figure 5.**
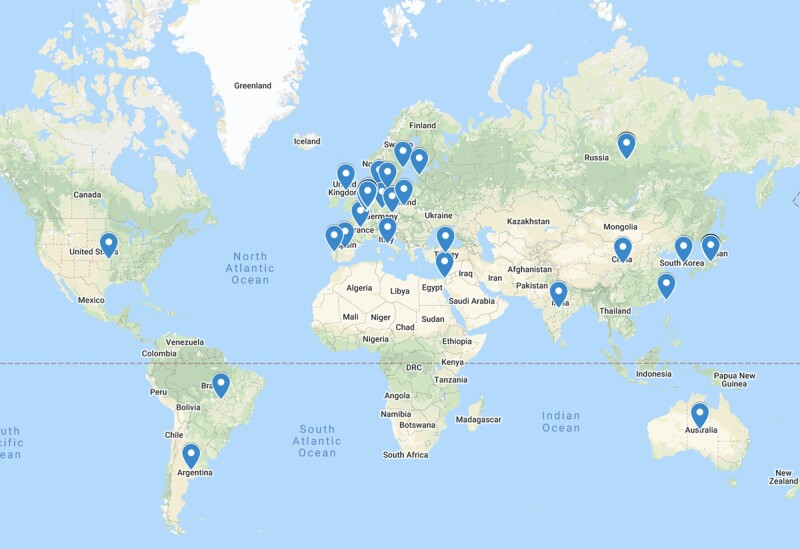
**Worldwide ovarian transplantation activity based on cases reported in the literature.** The blue pins represent the countries that have specialized centres offering ovarian tissue transplantation. The number of ovarian transplant cases in various countries that are published are as follows: Argentina (1), Australia (37), Belgium (26), China (1), Denmark (80), Estonia (1), France (162), Germany (92), Holland (1), India (3), Israel (24), Italy (5), Japan (8), Korea (12), Poland (1), Portugal (1), Prague (2), Russia (13) Spain (71), Sweden (3), Taiwan (1), Turkey (3), UK (3), USA (38) and Multi centre collaborations (65).

The ovarian tissue cryopreservation and transplantation procedure was noted to be the most prominent in Europe, especially in Belgium, Denmark, France, Germany and Spain. A collaborative network of German-speaking countries called ‘Fertiprotekt’, which includes Germany, Switzerland and Austria, forms one of the largest databases of ovarian cryopreservation and transplantation procedures. Figure 5 displays the worldwide activity.

## Discussion

This meta-analysis includes data from 568 women at individual level, as well as study level, and suggests that ovarian reproductive and endocrine function could be restored using fresh or frozen–thawed ovarian transplantation. The pooled results show a significant decrease in FSH and LH, and an increase in oestrogen post ovarian transplant. The median time to return of FSH to a value of <25 IU/l was 19 weeks, that of LH to a value of <15 IU/l was 19.5 weeks and menstruation was 18 weeks. A total 189 live births were reported in this systematic review. Two recent cohort studies that included 67 participants reported >50% pregnancy rates and more than 40% live birth rates (Hoekman *et al*., 2020; Shapira *et al.*, 2020). Our IPD meta-analysis, however, showed that the live birth rate from frozen–thawed ovarian transplants is 28%, which is in keeping with a recent study published by Dolmans *et al.* (2021) and previous systematic reviews (Gellert *et al.*, 2018; Lotz *et al.*, 2019; Sheshpari *et al.*, 2019). The meta-analysis also showed that the live birth rates from fresh transplants was 45% (95% CI: 23–86%). However, it is not possible to comment on the difference between fresh and frozen transplants owing to the very small sample size of fresh transplants. Although more miscarriages are reported in the fresh transplant group, sample size is extremely small for a comparison with frozen–thawed transplants. There is limited evidence on whether cryodamage or loss of follicles post-transplantation result in less favourable oocytes being fertilized and hence the pregnancy ending in miscarriage. The evidence however suggests that the main concern when transplanting tissue is the loss of follicles caused by ischaemia when retransplanting and hence the delay in revascularization (Lee *et al.*, 2016). Further research is required to explore whether there is an increased risk of miscarriage from fresh ovarian transplants. Furthermore, pregnancy rates were noted to be higher in participants in whom ovarian tissue was cryopreserved at ≤35 years. The results also suggest that higher concentrations of oestrogen are achieved when pelvic sites and more than one site is used for transplantation. Whether the ovarian tissue is transplanted onto the remaining ovary or pelvic peritoneal pockets, a similar number of live births was achieved (34 live births for transplant on to remaining ovary and 37 if transplanted at a close proximity to the ovary in a peritoneal pocket). It was estimated that ovarian grafts had a median duration of function of 2.5 years (IQR: 1.4–3.4, range: 0.7–5 years). This should be interpreted with caution, however, as the duration of graft function may correlate with the number of transplantations. Moreover, the procedure of ovarian tissue retrieval and transplantation is safe and the only risks associated are known intraoperative and postoperative complications for operative gynaecological laparoscopy and laparotomy (Beckmann *et al.*, 2017b). Likely variables such as the amount of the tissue transplanted and follicle density, which may influence oestrogen level, longevity of function and pregnancy, could not be assessed owing to a lack of, or variable, information reported. We found that most centres used slow freezing as a method of ovarian tissue cryopreservation with only 13 cases reported using vitrification. Evidence of pregnancies being achieved shows that vitrification could be considered as a method of cryopreserving ovarian tissue. A systematic review and meta-analysis conducted to assess the proportion of morphologically intact tissue after cryopreservation showed vitrification to be superior to slow freezing (Shi *et al.*, 2017). However, the protocols used for vitrification vary significantly and are not validated to support a change in practice. The data therefore needs to be interpreted with caution as further studies are required to draw definitive conclusions.

Although most centres will perform a unilateral oophorectomy for fertility preservation in women who require gonadotoxic anti-cancer therapies, through this review, we were not able to ascertain whether this alone puts young girls and women at risk of premature menopause (entering menopause before the age of 40 years). There is evidence to suggest that unilateral oophorectomy appears to reduce the age of menopause by 1–1.8 years (Cramer and Xu 1996; Yasui *et al.*, 2012; Bjelland *et al.*, 2014; Gasparri *et al.*, 2021). As for the reproductive outcomes in women who have undergone unilateral oophorectomy, recent evidence suggests that for unassisted reproduction, the outcomes were similar to those with two ovaries but when using ART, reduced live birth rates were reported. This is relevant and reassuring for healthy women who wish to preserve their fertility for social reasons, and who may be worried about their fertility if wishing to try naturally (Gasparri *et al.*, 2021). Chemotherapy before tissue retrieval, although reported in a small number of women having tissue transplanted, did not appear to compromise ability to conceive and achieve a live birth, which is very encouraging. This evidence is also supported by a recent study where the authors conclude that anti-cancer therapy before cryopreservation should not be considered a contraindication to opting for this method of fertility preservation (Shapira *et al.*, 2020). Many experts have commented on the possibility of recurrence of primary cancer or even emergence of new cancer in the ovarian graft (Dittrich *et al.*, 2015; Kristensen *et al.*, 2017). Although patients with cancers that have a high chance of recurrence should be treated with caution, in cases of recurrence, it is not always possible to prove that the cancer definitely originated in the transplanted graft.

When examining the type of cancer and related pregnancies and live births achieved, it was interesting to note that no pregnancies were achieved in the cervical cancer cohort: this in keeping with a recently published study in which no pregnancies were reported in this group either (Dolmans *et al.*, 2021). A study conducted by Anderson *et al.* (2018) showed that the pregnancy rates were noted to be the lowest in women suffering from cervical cancer as compared to other cancers (standardized incidence ratio = 0.34, 95% CI: 0.31–0.37). Cervical cancer affects relatively young women and there has been a steep increase in the incidence of cervical cancer in recent years (Arbyn *et al.*, 2020). Fertility preservation in females with cervical cancer is usually achieved by surgery (radical trachelectomy) but is only offered to those with early-stage disease, a good prognosis and ideally those who do not require adjuvant anti-cancer therapy in addition to surgery (Bentivegna *et al.*, 2016). This limits the candidature for fertility preservation in this cohort. Furthermore, there is evidence to suggest that treatment for cervical cancer (radiotherapy or chemotherapy) compromises uterine function as most women would need to undergo radiotherapy involving the uterus, resulting in fibrosis and scarring (Arbyn *et al.*, 2020; Teh *et al.*, 2014). There is also evidence to suggest that the dose and site of radiation (pelvic or total body) significantly impacts pregnancy outcomes (Teh *et al.*, 2014). Women who have had a fertility-sparing surgery may therefore still not be able to conceive (Somigliana *et al.*, 2020). Offering fertility preservation in this cohort needs to be carefully considered as they may not be able to conceive despite standard methods of fertility preservation before treatment. The return of hormonal function in this cohort, however, is promising and perhaps ovarian tissue cryopreservation could be considered to preserve hormonal function in these women. Furthermore, there is much debate as to whether AMH is valuable in predicting pregnancy and live birth rates in women undergoing OTC. Our data suggest that having a low AMH pre-transplantation does not predict a poor reproductive outcome in young girls and women who want to consider ovarian tissue cryopreservation as a method of fertility preservation. Ideally, one needs to measure AMH pre-retrieval, pre-transplant and at various time points post-transplant to be able to accurately predict pregnancy and live birth rates. Another factor to consider is the assay used for analysing the blood test, which adds further confounding to the results. For the reasons mentioned, the predictability of reproductive outcomes based on AMH alone should be interpreted with caution.

Additionally, through this review, we found that more pregnancies were achieved naturally as compared to using ART. Furthermore, recent studies have shown promising results from IVM of immature oocytes in ovarian cortical grafts. A study conducted on ovarian cortical tissue from 25 women demonstrated an unexpectedly high number of metaphase II oocytes being generated without stimulation (Nikiforov *et al.*, 2020). This gives further hope to many young girls and women who cannot undergo ovarian stimulation before chemotherapy, for achieving motherhood from ovarian tissue cryopreservation and transplantation.

Ovarian tissue cryopreservation and transplantation may still be deemed as experimental by some centres; however, many experts are now considering the potential use of this procedure in clinical practice and offering it as a routine fertility preservation method (Donnez *et al.*, 2013; Donnez *et al.*, 2015; Practice Committee of the American Society for Reproductive Medicine (ASRM), 2019). Evidence suggests that ovarian tissue cryopreservation may be particularly applicable to pre-pubertal girls and those at high risk of POI who require immediate gonadotoxic anti-cancer therapy and cannot wait for oocyte retrieval (Lambertini *et al.*, 2016; Matthews *et al.*, 2018). As per recommendation by the American Society for Reproductive Medicine, ovarian tissue cryopreservation is an acceptable technique for preserving fertility and is no longer considered experimental (ASRM, 2019). A fertility preservation guideline recently published by the ESHRE also recommends offering ovarian tissue cryopreservation in patients undergoing moderate- to high-risk gonadotoxic treatment, if patient prefers this method over oocyte and embryo freezing (European Society of Human Reproduction and Embryology (ESHRE), 2020). A guideline by the British Fertility Society (BFS) on fertility preservation for medical reasons in females describes the limitations of this technique when applied to adolescents and children. With increasing evidence of live births and return of endocrine function, however, the BFS concludes that ovarian tissue freezing should be considered in pre-pubertal girls (Lambertini *et al.*, 2016; Yasmin *et al.*, 2018). The BFS committee also agreed on the advantages of this technique with regards to the possibility of natural conception (especially if the ovarian tissue is transplanted in the pelvis, close to the fallopian tube) and proposed its use in post-pubertal patients, especially if oocyte cryopreservation is not possible. Furthermore, the possibility of continued hormone production from these transplants in order to prevent menopause has been emphasized by various experts in the literature (Donnez and Dolmans 2018; Yasmin *et al.*, 2018; Andersen *et al.*, 2019). Therefore ovarian transplants could also potentially be used as cell-based hormonal replacement therapy.

### Strengths

We performed a comprehensive search of the literature and synthesized the evidence from all studies available in this systematic review. While previous reviews have shed light on the outcomes of this procedure in the form of reproductive or hormonal function, the results have mainly been restricted to frozen–thawed transplants. This is the first systematic review that included outcomes from both fresh, frozen and donor ovarian tissue transplantation using unique IPD meta-analysis. Through a broad literature search, we identified all the reported ovarian transplant cases in addition to adding participant-level data through collaboration with centres that are established in providing this procedure. We also considered grey literature that includes an account of unsuccessful cases in various centres, conference proceedings and unpublished cases. This allowed inclusion of 87 studies and 735 women. To our knowledge, this is the largest collation of ovarian tissue transplantation outcomes to date. Our meta-analysis model can be updated, as such having the potential to create a worldwide network of ovarian cryopreservation and transplantation activity.

Furthermore, various relevant outcomes that determine reproductive and endocrine function, such as age at cryopreservation and transplantation technique, were analysed in addition to the time of return of hormonal function to premenopausal levels. We were also able to collate the outcomes based on disease, chemotherapy prior to tissue retrieval and site of transplant.

### Limitations

Most of the studies included small numbers of participants, which reduces our confidence in true success rates of this procedure. In some studies, hormonal function was described as being assessed immediately before tissue retrieval, whereas others assessed on the day of transplant, just prior to the procedure. For studies that did not explicitly describe the timing of hormonal tests, it is not possible to assess accurately the premenopausal status before the transplant. Also, in women having a remaining ovary, albeit menopausal, there is uncertainty regarding residual hormonal function from that ovary. Many studies also failed to mention the longevity of the graft to give an accurate estimate of the hormonal lifespan of the ovarian tissue. We were also unable to calculate the time to pregnancy. Not all pregnancies were conceived naturally and so it was not possible to predict how long it would have taken to conceive naturally. Through this review, 45 cases of fresh ovarian transplants were identified, but meta-analysis was only possible for two studies with a very small sample size (four women). Although the results were reassuring in terms of decreasing FSH to a level of <25 IU/l and levels of oestrogen increasing to >200 pmol/l, it was not possible to make a comparison with frozen–thawed transplants owing to such a small sample size.

Furthermore, clinical heterogeneity of the studies included in the meta-analysis resulted in weakness in our analysis. To overcome this, we endeavoured to gather IPD from authors and six centres agreed to provide this and further clarification. Collecting IPD was one of the significant challenges of this project. In order to help authors who may have a lack of time, funding or organizational support, we offered assistance in data collation and also sent them an outcome spreadsheet to assist with data collection (Nevitt *et al.*, 2017). From the 10 authors and centres that did not share data, six did not respond to emails despite reminders and four centres informed us that they were awaiting further publications or were simply not keen on collaboration despite a positive initial response. A scoping review that involved assessing the outcomes of IPD requests found that for academic studies eligible for IPD requests, only 33% provided the data (Ventresca *et al.*, 2020). We have managed to acquire data from 38% of the centres. Furthermore, we had requested data for 493 participants, from centres that had previously reported cohorts of five or more patients: we received data for 220 patients from six centres, which gives us a response rate of 45%. But despite the novelty of IPD meta-analysis on this topic, it has its limitations. When more than one publication originated from the same centre, population or cohort, there was a possibility for double counting the patients. For this reason, reports were individually assessed by two reviewers independently to identify and remove duplicates. This was also double-checked by cross referencing and contacting the authors directly for clarification, and with the most recent or complete publication being selected. To ensure that the IPD and aggregate samples were a random sample from the population of interest, we compared the studies and conducted a meta-analysis of reproductive outcomes. We found that the live birth rate for studies that provided aggregate data was 28% (95% CI: 23–35%) compared to that for IPD, which was 30% (95% CI: 23–38%). This supports the assumption that the data are missing at random and the ones that did not provide IPD are not systematically different from the ones that provided it (Supplementary Figs S9 and S10 and Supplementary Table SIX). Finally, the ovarian transplant procedure is carried out worldwide in different centres with variable protocols and some degree of heterogeneity is to be expected. This review also highlights how this procedure is currently provided, showing lack of consensus in the delivery of this technique as well as an urgent unmet need for a worldwide registry.

### Implications for clinical practice

Through previous publications and this review, we can conclude that ovarian tissue transplantation has shown promising results in preserving ovarian reproductive and endocrine function. This procedure should no longer be considered experimental and it should be offered to women who wish to preserve their fertility out of the research context. The potential use of transplants in preserving hormonal function is an area that needs to be explored further. There is currently no robust guidance for clinicians or patients about this procedure and its potential uses. With its potential use in alleviating menopausal symptoms and improving quality of life, there is an urgent unmet need for a robust policy and standard. The results of this review will hopefully guide clinicians in advising women about the benefits and shortcomings of this procedure until a formal guideline is produced.

### Implications for further research

Further data with larger studies that include participants with results that were not successful need to be included to give an accurate success rate of this procedure. Furthermore, a robust guideline for the optimal size of the tissue graft and surgical procedure for retrieval and transplantation of ovarian tissue needs to be produced to guide centres with this technique. There is also an urgent unmet need for a worldwide registry to provide a database that records all cases of ovarian transplantation. Finally, predefined outcome sets need to be determined to ensure all randomized control trials, cohort studies or case series report similar outcomes for ovarian transplantation.

## Supplementary data

Supplementary data are available at *Human Reproduction Update* online.

## Data availability 

The data underlying this article are available in the article and its online supplementary material.

## Supplementary Material

dmac003_Supplementary_Data
