# Supplementary material for: Fresh and cryopreserved ovarian tissue transplantation for preserving reproductive and endocrine function: a systematic review and individual patient data meta-analysis
Source: Hum Reprod Update. 2022 Feb 24;28(3):400–16. doi: 10.1093/humupd/dmac003 (PMC9733829; doi:10.1093/humupd/dmac003)
Supplement: dmac003_Supplementary_Data [file dmac003_supplementary_data.zip › dmac003-suppl_data/Supplementary figures final EO.pptx]

## Slide 1
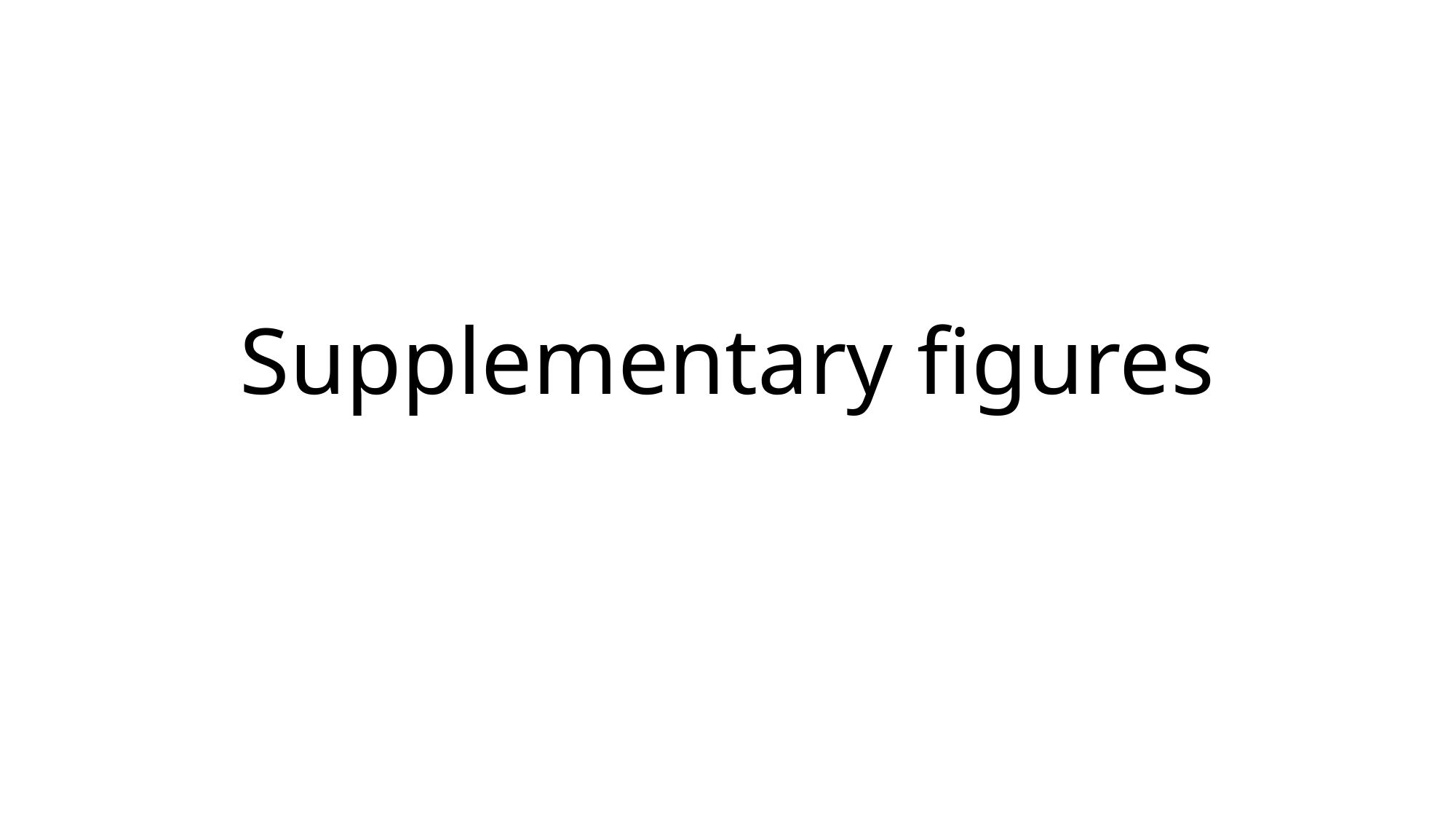

# Supplementary figures

## Slide 2
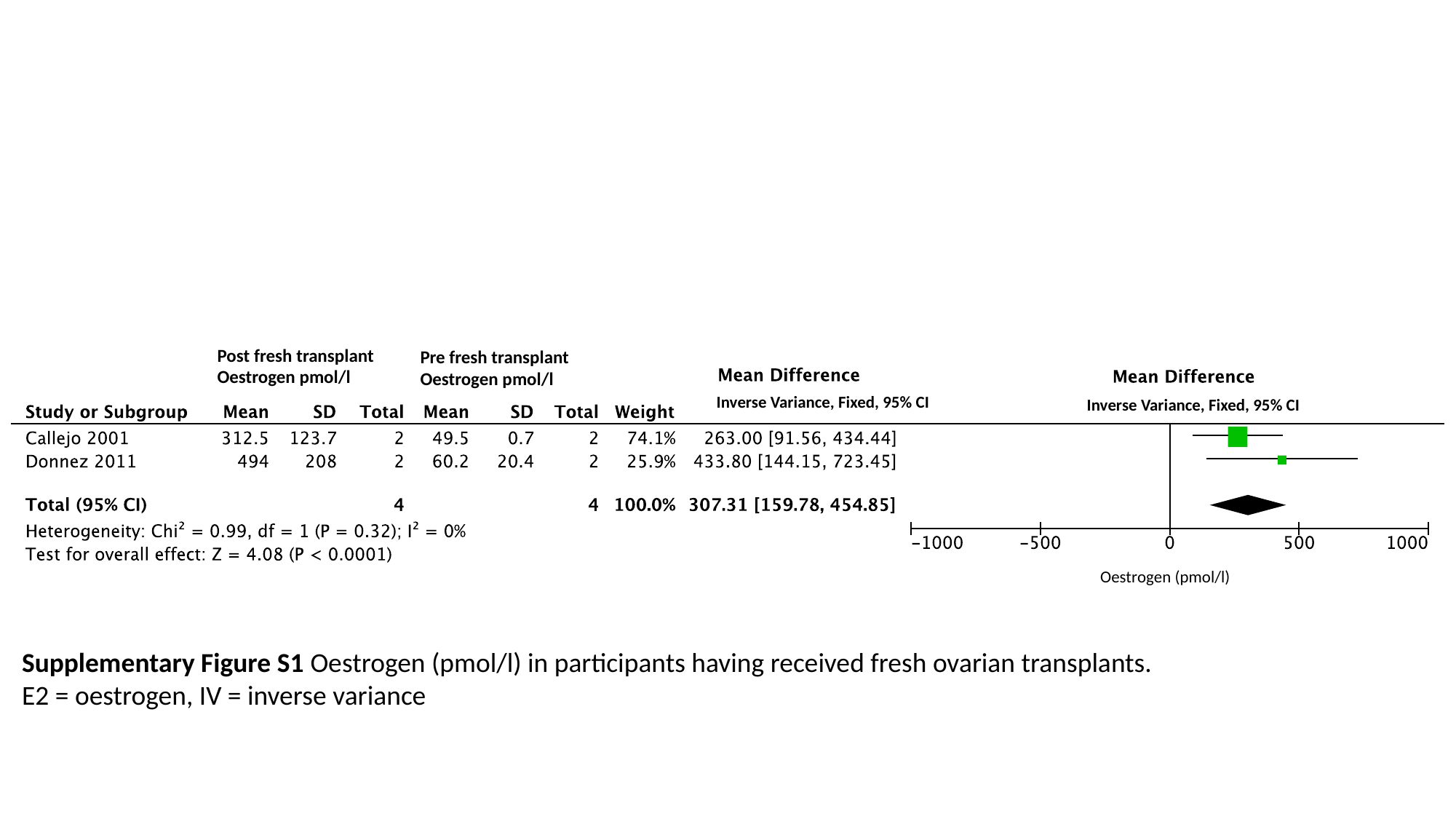

Post fresh transplant
Oestrogen pmol/l
Pre fresh transplant
Oestrogen pmol/l
Inverse Variance, Fixed, 95% CI
Inverse Variance, Fixed, 95% CI
Oestrogen (pmol/l)
Supplementary Figure S1 Oestrogen (pmol/l) in participants having received fresh ovarian transplants.
E2 = oestrogen, IV = inverse variance

## Slide 3
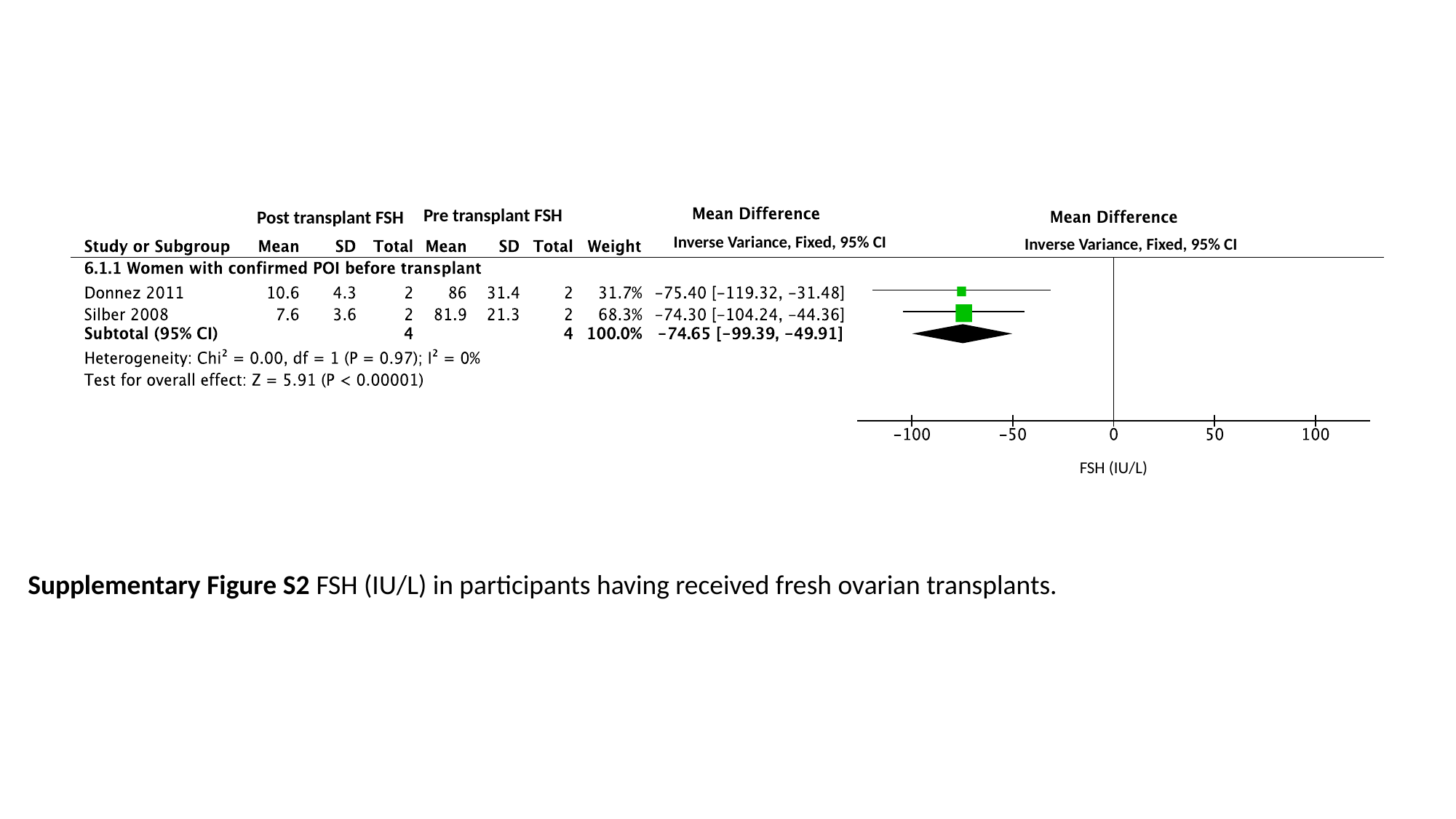

Pre transplant FSH
Post transplant FSH
Inverse Variance, Fixed, 95% CI
Inverse Variance, Fixed, 95% CI
FSH (IU/L)
Supplementary Figure S2 FSH (IU/L) in participants having received fresh ovarian transplants.

## Slide 4
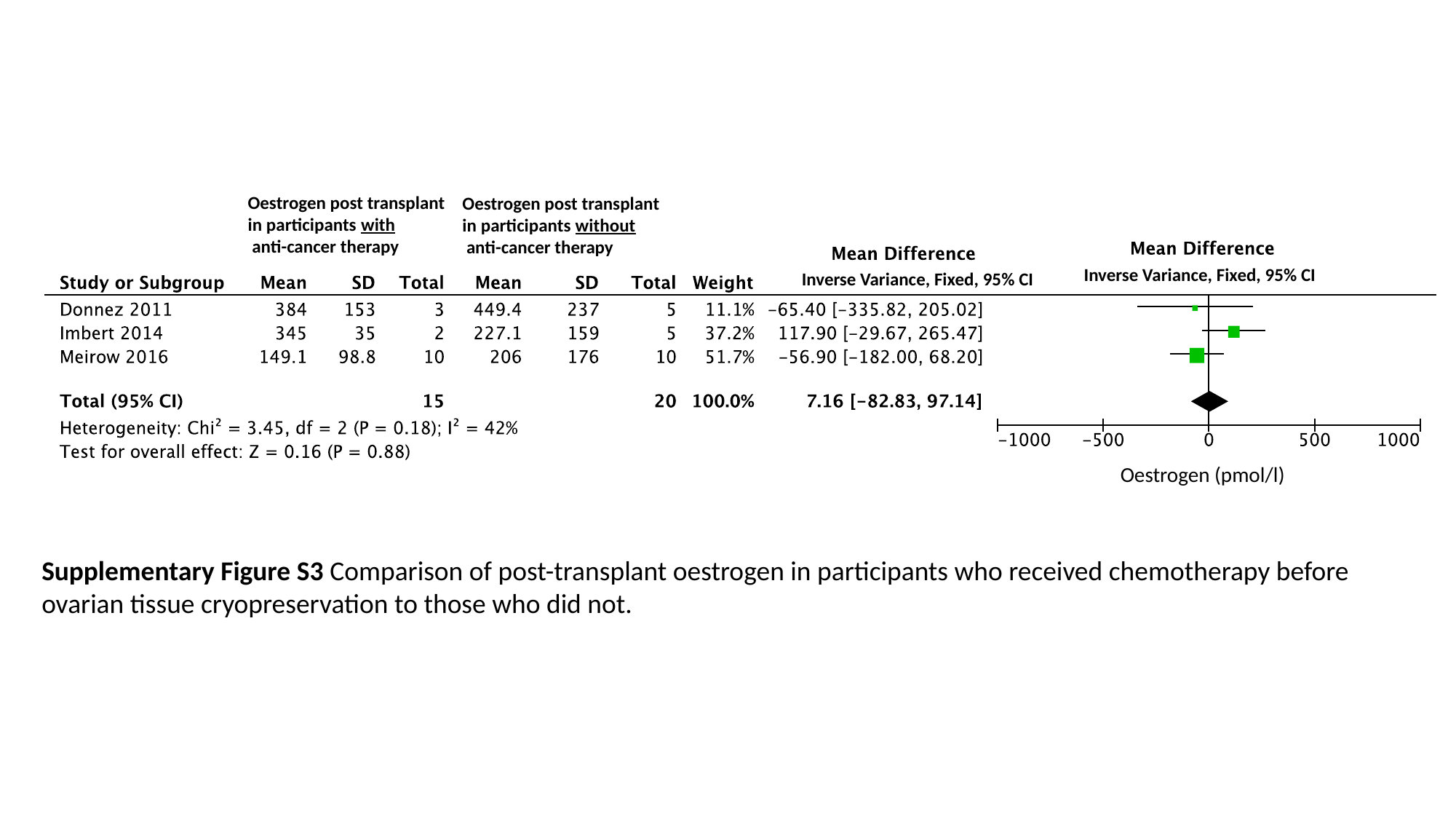

Oestrogen post transplant
in participants with
 anti-cancer therapy
Oestrogen post transplant
in participants without
 anti-cancer therapy
Inverse Variance, Fixed, 95% CI
Inverse Variance, Fixed, 95% CI
Oestrogen (pmol/l)
Supplementary Figure S3 Comparison of post-transplant oestrogen in participants who received chemotherapy before ovarian tissue cryopreservation to those who did not.

## Slide 5
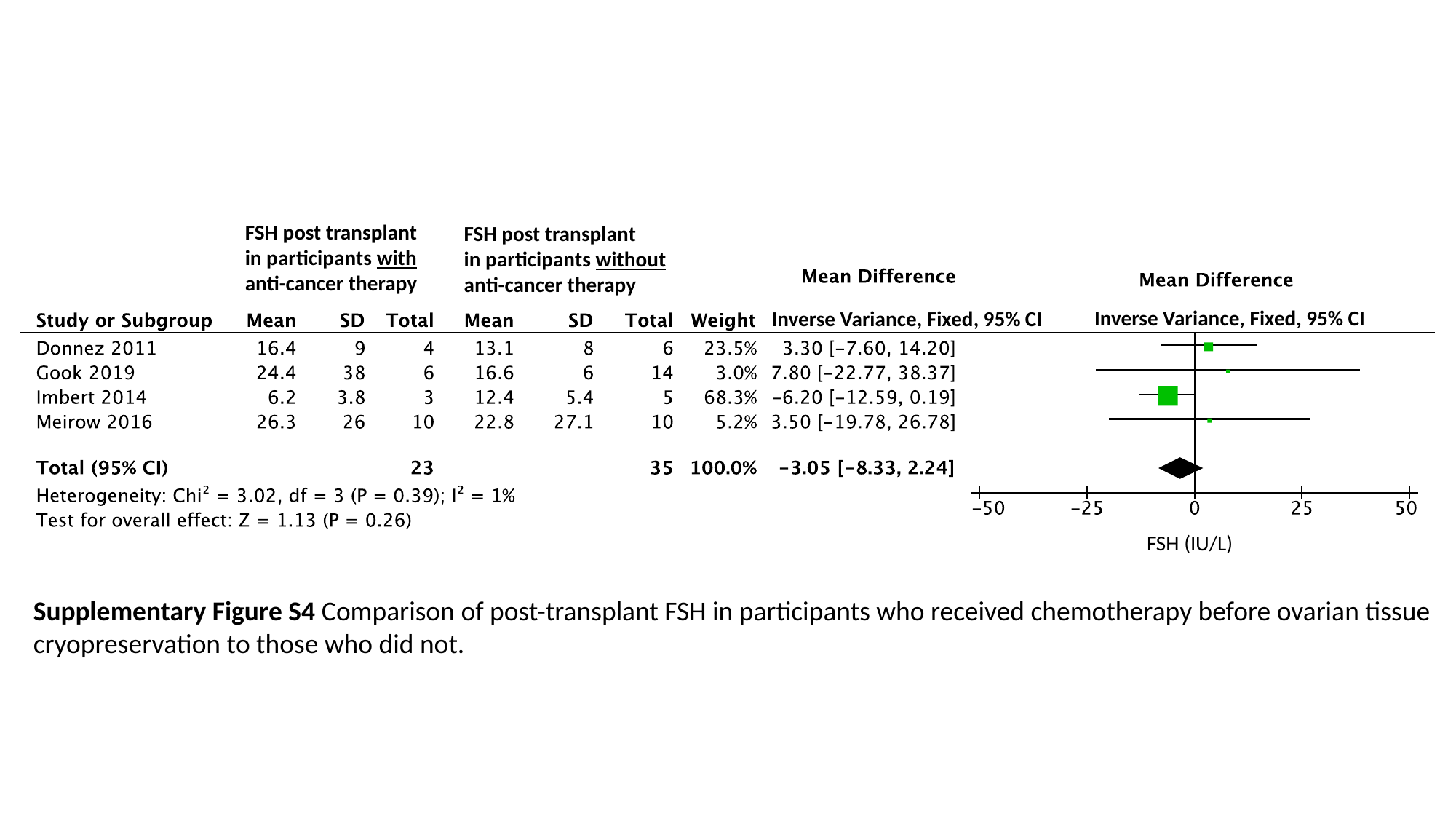

FSH post transplant
in participants with
anti-cancer therapy
FSH post transplant
in participants without
anti-cancer therapy
Inverse Variance, Fixed, 95% CI
Inverse Variance, Fixed, 95% CI
FSH (IU/L)
Supplementary Figure S4 Comparison of post-transplant FSH in participants who received chemotherapy before ovarian tissue cryopreservation to those who did not.

## Slide 6
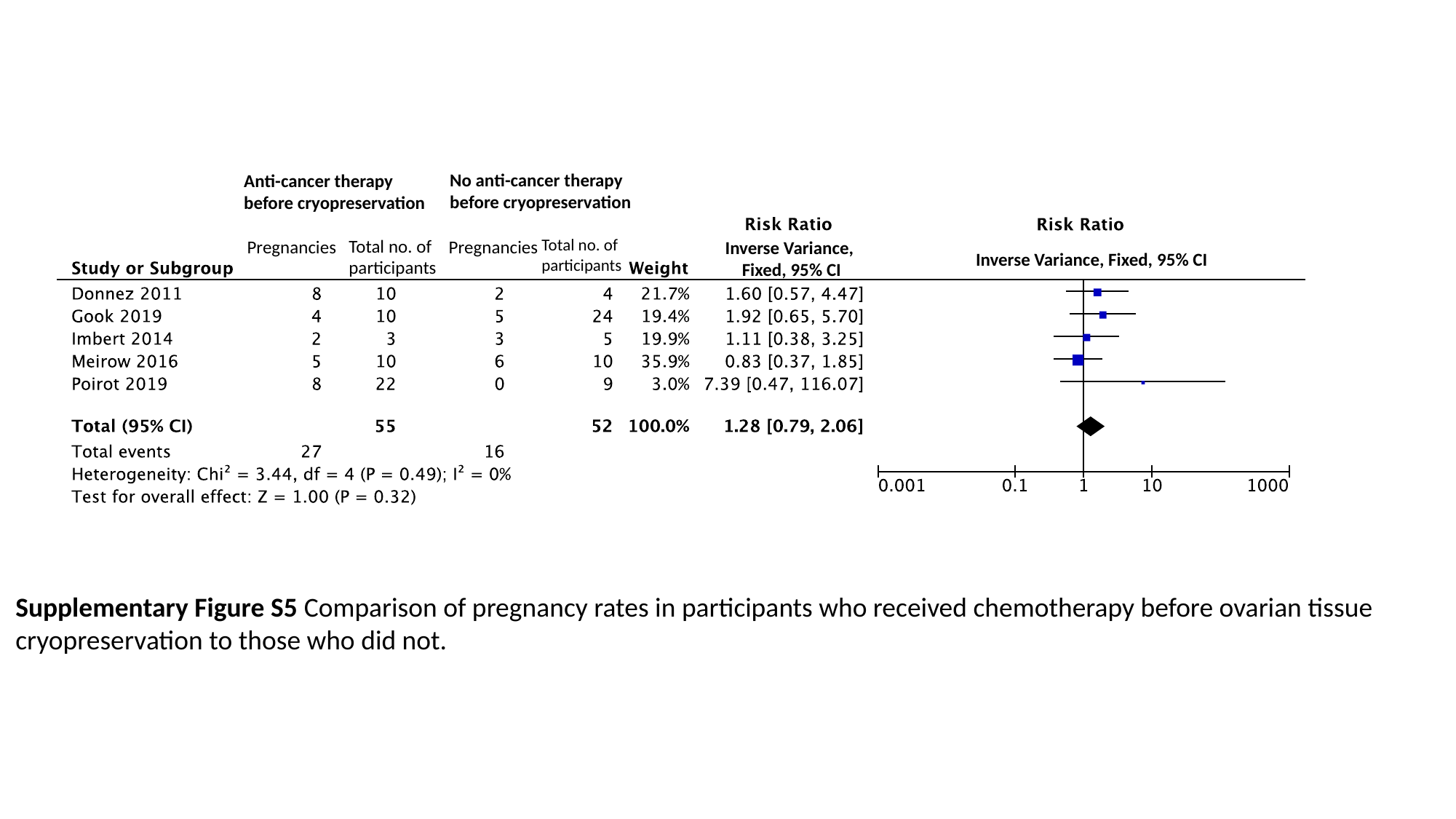

No anti-cancer therapy
before cryopreservation
Anti-cancer therapy
before cryopreservation
Total no. of
participants
Total no. of
participants
Pregnancies
Pregnancies
Inverse Variance,
Fixed, 95% CI
Inverse Variance, Fixed, 95% CI
Supplementary Figure S5 Comparison of pregnancy rates in participants who received chemotherapy before ovarian tissue cryopreservation to those who did not.

## Slide 7
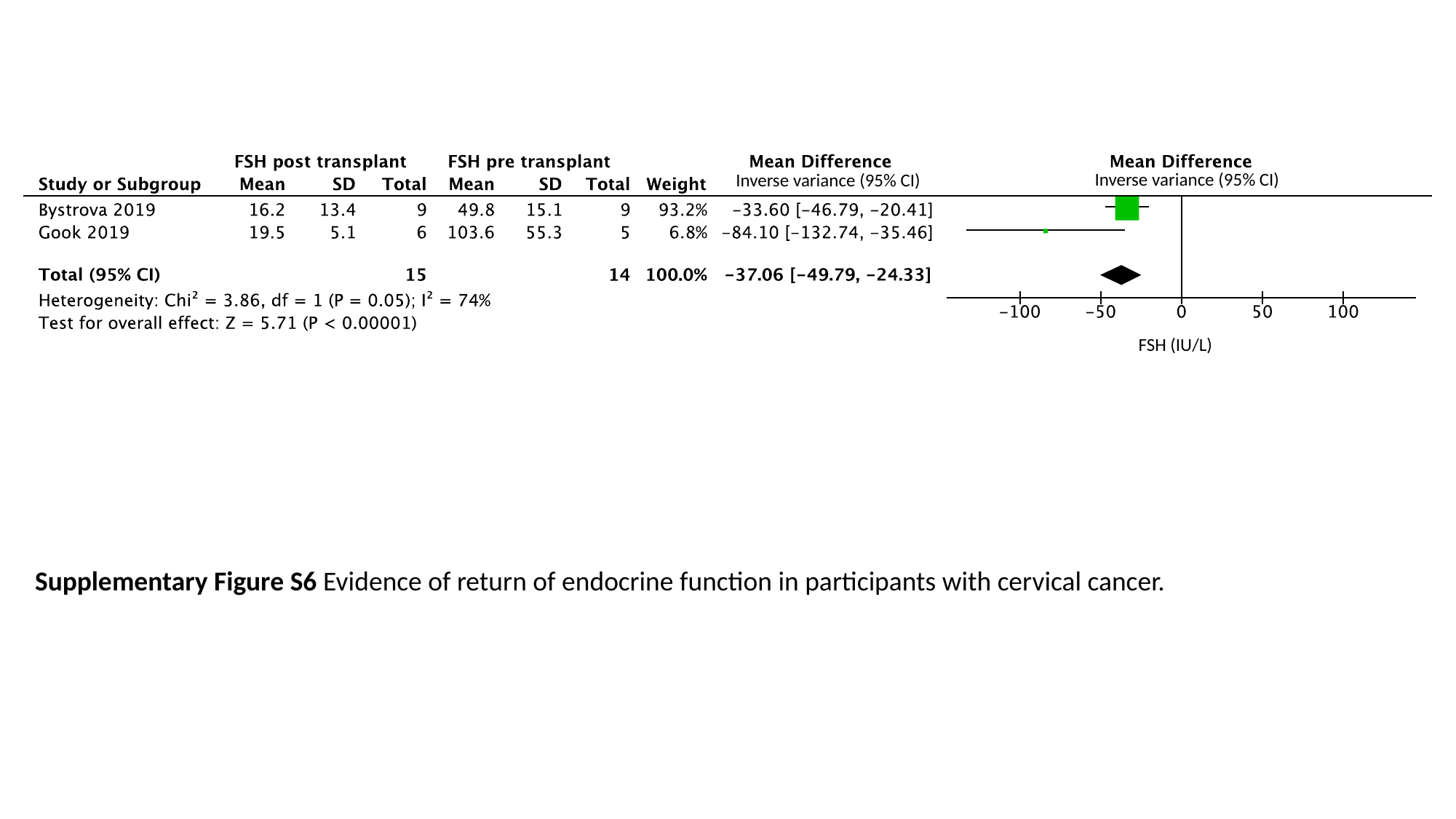

Inverse variance (95% CI)
Inverse variance (95% CI)
FSH (IU/L)
Supplementary Figure S6 Evidence of return of endocrine function in participants with cervical cancer.

## Slide 8
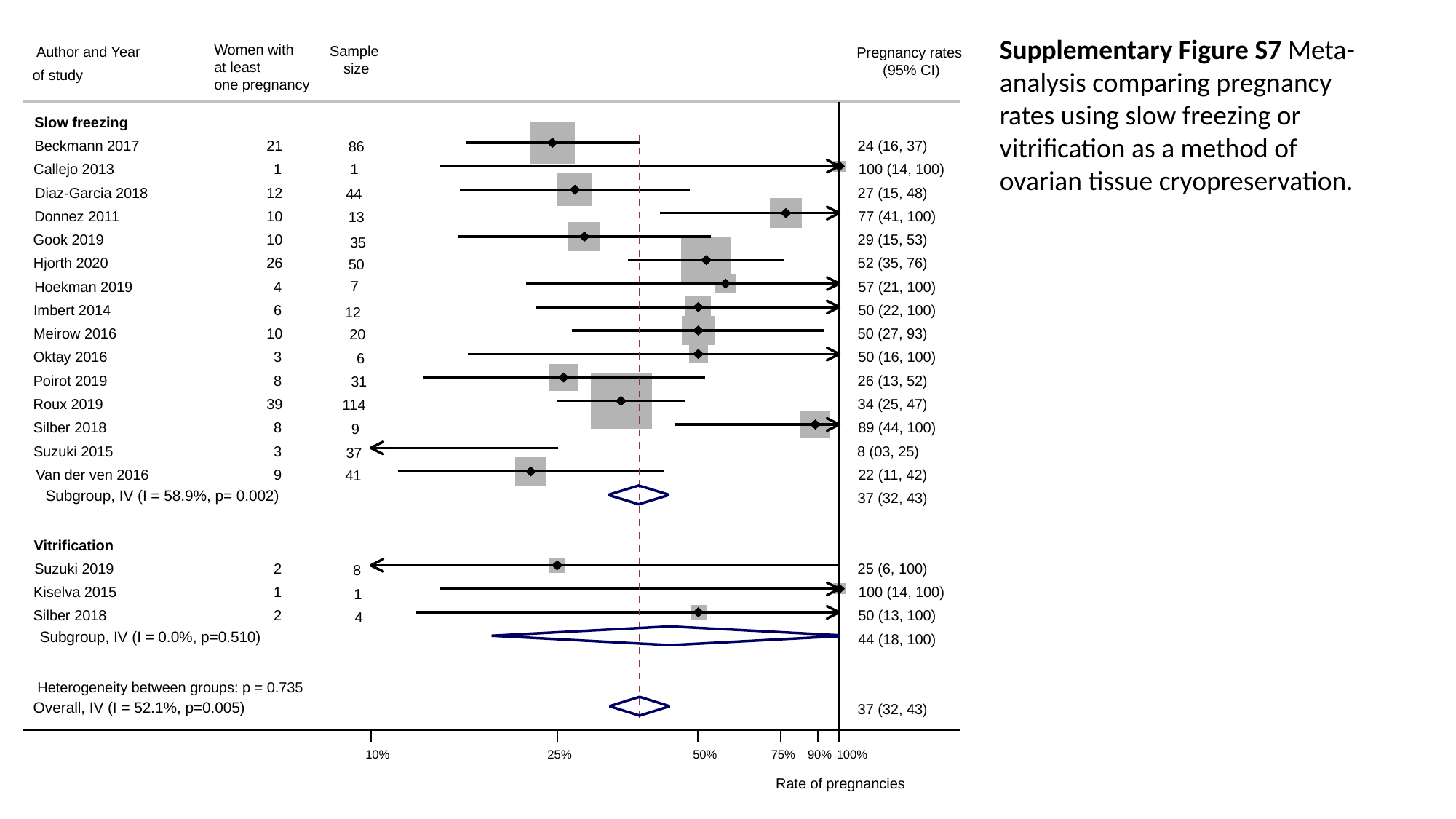

Women with
at least
one pregnancy
Sample
size
Author and Year
Pregnancy rates
 (95% CI)
of study
Slow freezing
Beckmann 2017
21
24 (16, 37)
86
Callejo 2013
1
100 (14, 100)
1
Diaz-Garcia 2018
12
27 (15, 48)
44
Donnez 2011
10
77 (41, 100)
13
Gook 2019
10
29 (15, 53)
35
Hjorth 2020
26
52 (35, 76)
50
7
Hoekman 2019
4
57 (21, 100)
Imbert 2014
6
50 (22, 100)
12
Meirow 2016
10
50 (27, 93)
20
Oktay 2016
3
50 (16, 100)
6
Poirot 2019
8
26 (13, 52)
31
Roux 2019
39
34 (25, 47)
114
Silber 2018
8
89 (44, 100)
9
Suzuki 2015
3
8 (03, 25)
37
Van der ven 2016
9
22 (11, 42)
41
Subgroup, IV (I = 58.9%, p= 0.002)
37 (32, 43)
Vitrification
Suzuki 2019
2
25 (6, 100)
8
Kiselva 2015
1
100 (14, 100)
1
Silber 2018
2
50 (13, 100)
4
Subgroup, IV (I = 0.0%, p=0.510)
44 (18, 100)
Heterogeneity between groups: p = 0.735
Overall, IV (I = 52.1%, p=0.005)
37 (32, 43)
90%
10%
25%
50%
75%
100%
Rate of pregnancies
Supplementary Figure S7 Meta-analysis comparing pregnancy rates using slow freezing or vitrification as a method of ovarian tissue cryopreservation.

## Slide 9
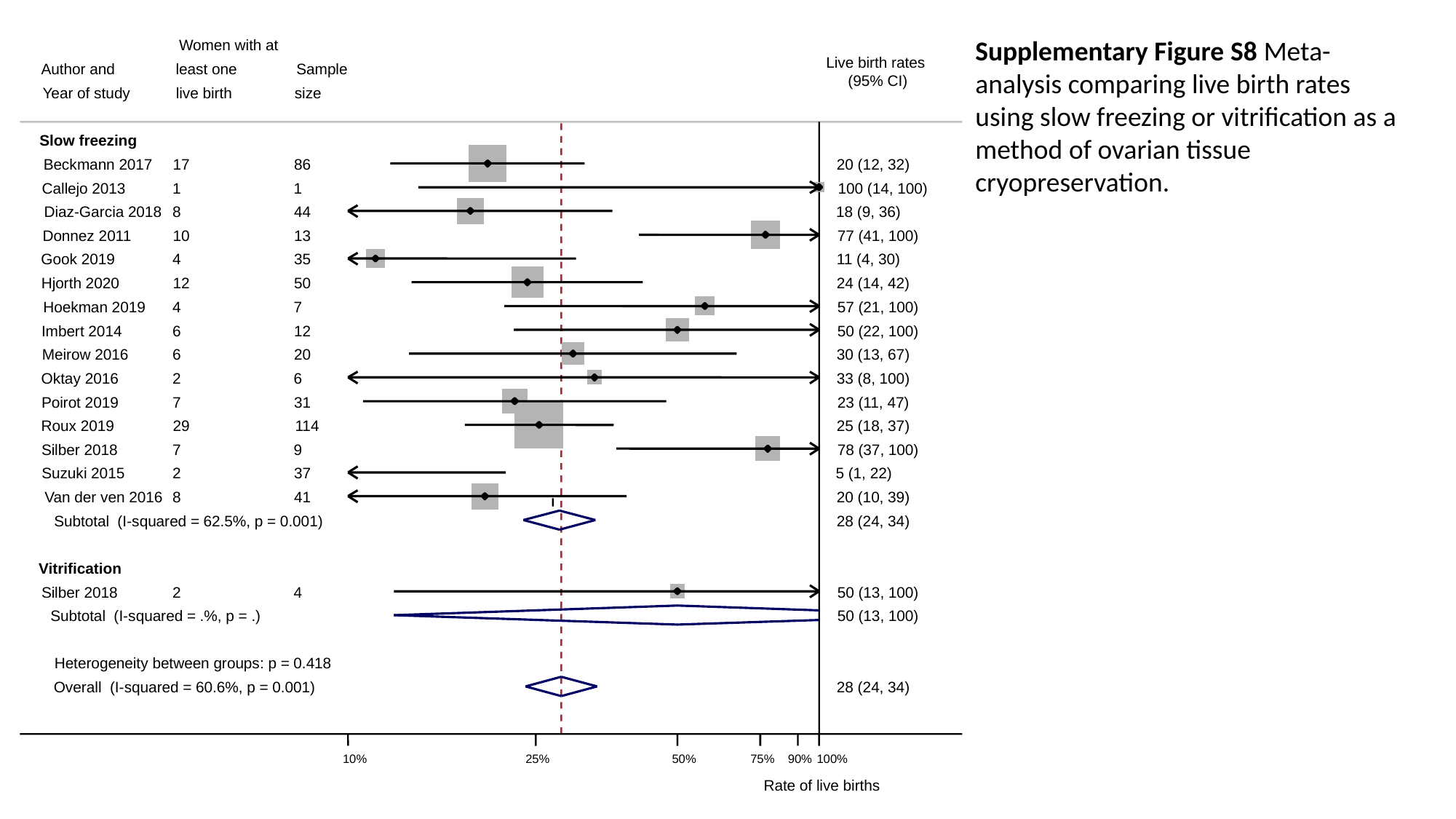

Women with at
Live birth rates
 (95% CI)
Author and
least one
Sample
Year of study
live birth
size
Slow freezing
Beckmann 2017
17
86
20 (12, 32)
Callejo 2013
1
1
100 (14, 100)
Diaz-Garcia 2018
8
44
18 (9, 36)
Donnez 2011
10
13
77 (41, 100)
Gook 2019
4
35
11 (4, 30)
Hjorth 2020
12
50
Hoekman 2019
4
7
57 (21, 100)
Imbert 2014
6
12
50 (22, 100)
Meirow 2016
6
20
30 (13, 67)
Oktay 2016
2
6
33 (8, 100)
Poirot 2019
7
31
23 (11, 47)
Roux 2019
29
114
25 (18, 37)
Silber 2018
7
9
78 (37, 100)
Suzuki 2015
2
37
5 (1, 22)
Van der ven 2016
8
41
20 (10, 39)
Subtotal (I-squared = 62.5%, p = 0.001)
28 (24, 34)
Vitrification
Silber 2018
2
4
50 (13, 100)
Subtotal (I-squared = .%, p = .)
Heterogeneity between groups: p = 0.418
Overall (I-squared = 60.6%, p = 0.001)
28 (24, 34)
24 (14, 42)
50 (13, 100)
10%
25%
50%
75%
90%
100%
Rate of live births
Supplementary Figure S8 Meta-analysis comparing live birth rates using slow freezing or vitrification as a method of ovarian tissue cryopreservation.

## Slide 10
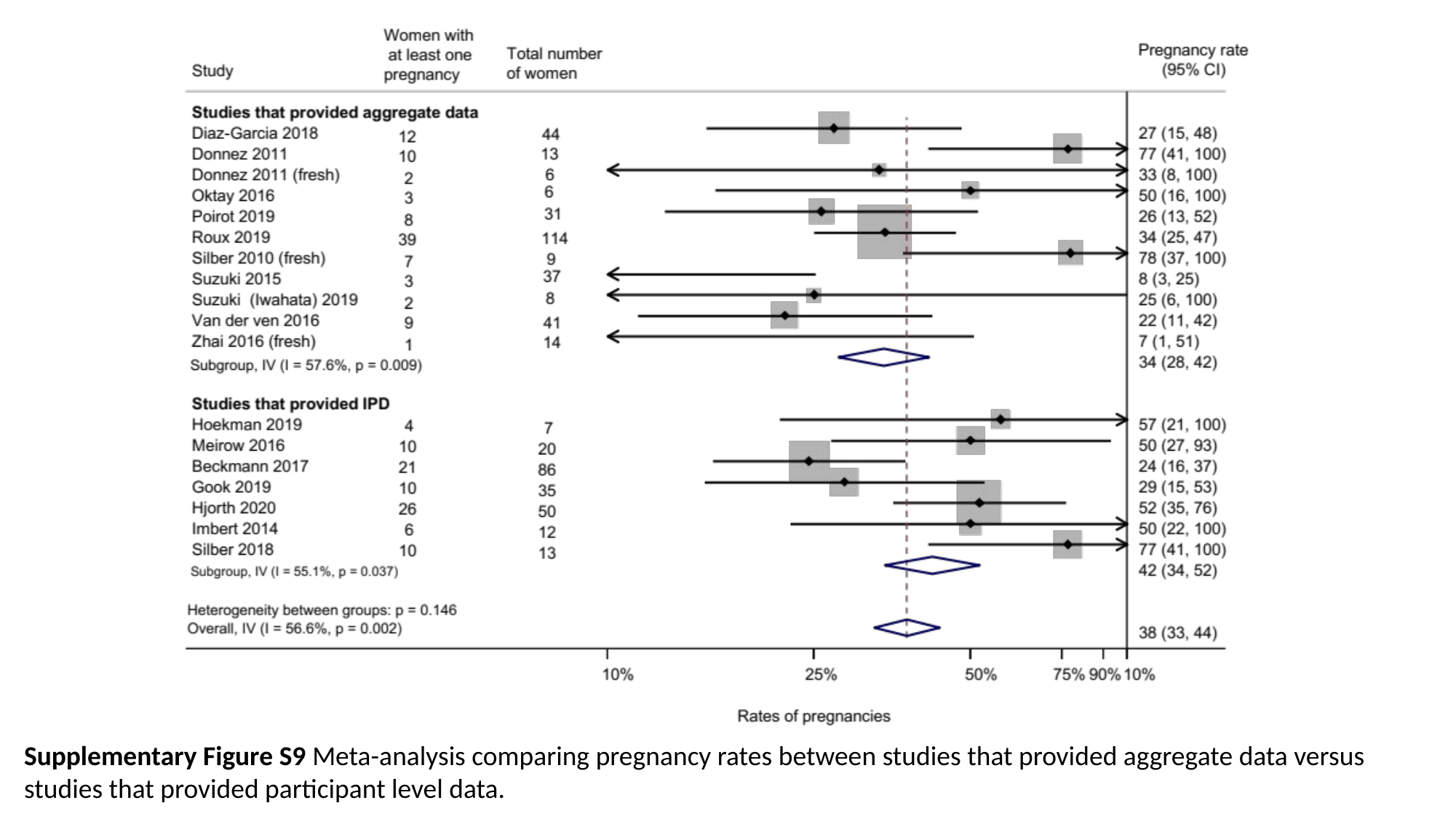

Supplementary Figure S9 Meta-analysis comparing pregnancy rates between studies that provided aggregate data versus studies that provided participant level data.

## Slide 11
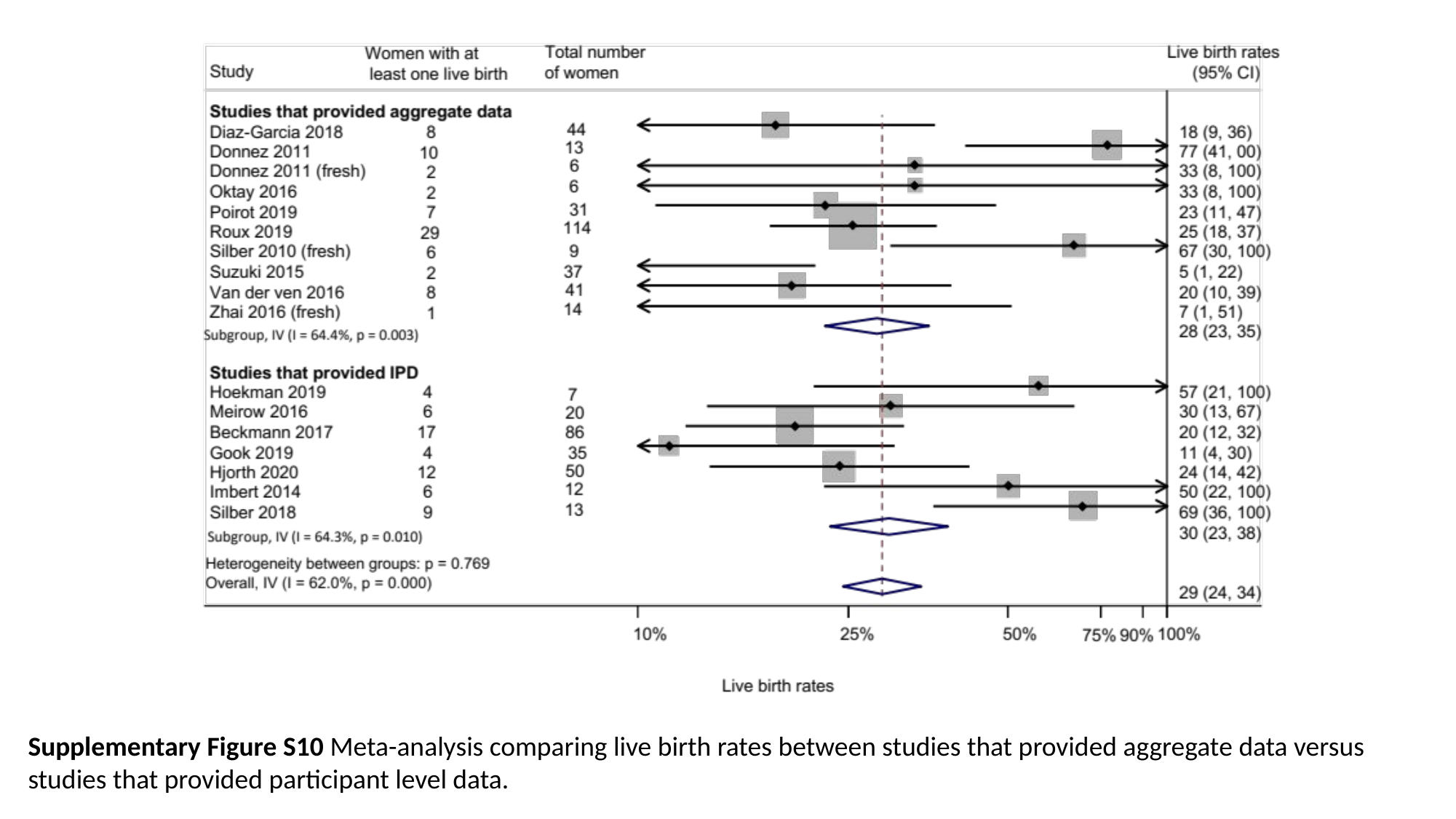

Supplementary Figure S10 Meta-analysis comparing live birth rates between studies that provided aggregate data versus studies that provided participant level data.
