# Supplementary material for: Fresh and cryopreserved ovarian tissue transplantation for preserving reproductive and endocrine function: a systematic review and individual patient data meta-analysis
Source: Hum Reprod Update. 2022 Feb 24;28(3):400–16. doi: 10.1093/humupd/dmac003 (PMC9733829; doi:10.1093/humupd/dmac003)
Supplement: dmac003_Supplementary_Data [file dmac003_supplementary_data.zip › dmac003-suppl_data/Supplementary Table II (study characteristics table).docx]

**Supplementary Table 2. Study characteristics for patients who received ovarian transplantation.**

| **References**  **(Country of origin)** | **Women undergoing fertility preservation (n)** | **Age (Mean, SD)** Cryopreservation  Transplantation | **Type of ovarian transplants (Fresh or frozen, n, size of transplant)** | **Surgical technique** | | **Reproductive and endocrine outcomes + follow up reported** |
| --- | --- | --- | --- | --- | --- | --- |
|  |  |  |  | **Surgical approach for transplantation (n)** | **Site of transplant (n)** |  |
| **Case series/Cohort studies**  **(Studies with ≥ 5 patients)** | | | | | | |
| ***Beckmann et al. 2017a****, Beckmann et al. 2017b+ IPD unpublished data^6^  (Germany) | AML (1),  Anal carcinoma (5),  Anti-Synthetase-Syndrome (1),  Breast cancer (28),  cervical cancer (3),  CML (3),  Dysgerminoma (1),  endometrial cancer (1),  HL (29),  Ovarian cancer (7),  POI (3),  Rectal cancer (3),  SLE (1),  social freezing (1) | **Cryopreservation**  30.5 (5.4)  **Transplantation**  35.3 (4.9) | Frozen (86)  Amount of tissue transplanted not described  (Size 3x2x1mm) | Laparoscopy (86) | Remaining ovary + Pelvic side wall (4)  Pelvic side wall (82) | FSH, LH and oestrogen at baseline and post-transplant  Return of menstrual activity  Pregnancies  Live births |
| Bystrova et al. 2019  (Russia) | Cervical cancer (9)  Endometrial Cancer (1) | **Cryopreservation** 30.8 (4.4)  **Transplantation**  31.8 (4.4) | Frozen (10)  10 fragments (5x5mm) | SC + Laparoscopy (3)  SC (7) | SC abdominal wall (6), SC forearm + abdominal wall (1), SC forearm (1), SC abdominal wall + pelvic peritoneum (2) | FSH and LH at baseline and post-transplant  Oestrogen post-transplant  3 year follow up |
| Callejo et al. 2001 and Callejo et al. 2013  (Spain) | TAH + BSO (4)  Dermoid cyst (1) | **Cryopreservation**  20^3^  **Transplantation**  44 (7.8) | Fresh (3)  Frozen (2)  Size of fragment transplanted (1x1x1 cm) | Laparoscopy (1), SC (2), IM (2) | SC arm (2),  IM Rectus abdominis muscle (2), Pelvic side wall (1) | FSH and Oestrogen at baseline and post-transplant  Return of menstruation  Pregnancies  Live births  1 year follow up |
| Diaz-Garcia et al. 2018 (Spain  ) | Breast cancer (31)  HL (9),  Rectal cancer (2),  Medulloblastoma (1)  Persistent trophoblastic disease (1) | **Cryopreservation**  34.3 (7.2)  **Transplantation**  38.9 (4.1) | Frozen (44) | Laparoscopy (1)  Laparotomy (41) | Subcortical pouches (24), Cortical microsurgical sutures (26),  Sub peritoneal pouches (27) | Pregnancies  Live births |
| ***Donnez et al. 2011**** (Donnez et al 2004; Donnez et al. 2005; Donnez et al. 2006; Donnez et al. 2007; Camboni et al. 2008; Donnez et al. 2008; Donnez and Squifflet 2010; Donnez et al. 2011; Donnez and Squifflet 2011)^4^  (Belgium) | AML (1)  Beta Thalassemia (1)  Breast cancer (1)  BSO (1)  HL (4)  MPA (1)  Neuroectodermal tumour of right orbit (1)  NHL (2)  Sickle cell anaemia (4)  Turner's syndrome (1) | **Cryopreservation**  24 (5.1)  **Transplantation**  29.7 (4.7) | Fresh (2)  Fresh donor (4)  Frozen (13)  Size of tissue not given | Mini laparotomy (4),  2 step laparoscopy (2)  Laparoscopy (3)  Laparotomy (2)  Not given (8) | Remaining ovary (10),  Previous ovary +Pelvic side wall (4),  Remaining ovary +SC (1),  Pelvic side wall (4) | FSH at baseline and post transplantation  Oestrogen at baseline and post-transplant  Return of menstruation  Pregnancies  Live births |
| Gook et al. 2019  (Stern et al. 2011, Stern et al. 2013; Stern et al. 2014) + IPD (unpublished data)  (Australia) | Autoimmune polyarteritis (1)  B-cell lymphoma (1)  Breast cancer (1)  Cervical cancer (9)  Cyst (1)  Desmoid Tumour (1)  Endometrial cancer (1)  Endometriosis (1)  Ewing's sarcoma (2)  Gynaecological Cancer (2)  HL (4)  NHL (3)  NMDA encephalitis (1)  Pelvic Sarcoma (1)  Sarcoma (1)  SLE (1)  Social freezing (2)  Wegner's (2) | **Cryopreservation**  29.1 (6.1)  **Transplantation**  35.5 (5.7) | Frozen (35)  Average amount of tissue transplant = 330.1 mm^3^ | Laparoscopy (35) | Remaining ovary + Pelvic side wall (21),  Pelvic side wall (14) | FSH and oestrogen at baseline and post-transplant  Pregnancies  Live births |
| Hoekman et al. 2019, Klijn et al. 2019  (The Netherlands) | Breast Cancer (2)  Hodgkin’s lymphoma (2)  Non-Hodgkin’s lymphoma (1)  Ewing’s sarcoma (1) | **Cryopreservation**  27 (4.7)  **Transplantation**  33.4 (5.8) | Frozen (7)  Average of 8 pieces transplants (10×5×1 mm) | Not reported | Remaining ovary (6)  Remaining ovary + peritoneal wall (1) | Return of menstruation  Pregnancies  Live births |
| ***Imbert et al. 2014**** (Demeestere et al. 2015 + IPD provided by author)  (Belgium) | Breast cancer (3)  Rectal cancer (1)  HL (3)  NHL (2)  Sarcoma (1)  Sickle cell anaemia (1)  Wegner's (1) | **Cryopreservation** 27.5 (5.8)  **Transplantation**  33.1 (5.1) | Frozen (12)  Amount and size of transplanted tissue not given | Not provided | Pelvic sidewall +SC (2),  Remaining ovary + Pelvic side wall (2), Remaining ovary + pelvic side wall + SC (1) | FSH and oestrogen at baseline and post-transplant  Return of menstruation  Pregnancies  Live births  5 months follow up |
| Janse et al. 2011  (Belgium and The Netherlands) | Sickle cell anaemia (2)  AML (1)  HL (1)  Wegner's (1)  NHL (1)  Endometriosis (1) | **Cryopreservation**  23 (5.4)  **Transplantation**  29.6 (3.6) | Frozen (6)  Fresh (1)  Size of transplant not given | Not reported | Remaining ovary (6),  Peritoneal window (1) | FSH at baseline and post-transplant  Oestrogen at baseline and post-transplant  Return of menstruation  Pregnancies  2.5 year follow up |
| ***Jensen et al. 2015****  (Schmidt et al. 2004; Schmidt et al. 2005; Rosendahl et al. 2006;  Andersen et al 2008; Schmidt et al. 2011; Andersen, C. Y., et al. 2012; Greve et al.2012; Ernst et al 2013; Macklon et al. 2014, Hjorth et al 2020)^1^  (Denmark) | Breast cancer (12)  HL (5)  NHL (5)  Cervical cancer (3)  Aplastic anaemia (2)  Ewing sarcoma (2) Paroxysmal nocturnal haemoglobinuria (2)  Sarcoma (2)  Haemolytic uraemic syndrome (1)  Ovarian cancer (1)  Colon cancer (1)  Anal cancer (1)  Autoimmune Small Vessel Vasculitis (1)  Morbus Behcet (1) Choriocarcinoma (1)  Wegener’s Granulomatosis (1) | **Cryopreservation** 30 (5.8)  **Transplantation**  33.7 (5.6) | Frozen (58)  (5x5x1mm) | Mini laparotomy (19)  Laparoscopy + mini laparotomy (24)  Laparoscopy (15) | Remaining ovary (19),  Remaining ovary + Peritoneal pocket (18),  Peritoneal pocket (15),  Remaining ovary + Abdominal wall (3)  Remaining ovary +Peritoneal pocket +Abdominal wall (3), | FSH at baseline and post-transplant  Oestrogen at baseline and post-transplant  Return of menstruation  Pregnancies  Live births  10 year follow up |
| Meirow et al. 2016  (Israel) | AML (1)  Breast Cancer (3)  CML (1)  Ewing's sarcoma (2)  HL (9)  NHL (4) | **Cryopreservation** 28.7 (7.5)  **Transplantation**  34.4 (6.9) | Frozen (20)  Amount of transplant not given. (5x10x1.5mm) | Mini laparotomy (20) | Remaining ovary (20) | FSH and oestrogen at  at baseline and post transplantation  Return of menstruation  Pregnancies  Live births  Follow up 7 to 141 months (mean 3.18 years) |
| ***Oktay et al. 2016****  (Oktay et el. 2003; Oktay et el 2004; Oktay et el 2011)  (USA) | Cervical cancer (1)  Benign ovarian cysts (1)  Breast cancer (1)  HL (1)  hemophagocytic lymphohistiocytosis (1)  NHL (1) | **Cryopreservation**  27 (4.7)  **Transplantation**  34.5 (2.4) | Frozen (4)  Fresh (2)  Amount of tissue transplanted not given.  (5x5x2 mm) | Laparoscopy (1)  Robotic-assisted laparoscopy (1)  SC (2)  IM (2) | Remaining ovary (2),  SC (2),  IM (2) | FSH at baseline and post-transplant  Oestrogen post-transplant  Return of menstrual activity  Pregnancies  Live births  2 year follow up |
| Poirot et al. 2019  (France) | Beta thalassemia (1)  Cervical cancer (1)  Ewing's sarcoma (1)  HL (14)  NHL (8)  Ovarian cancer (3)  Pseudomyxomal peritonei (1)  Shwachman-Diamond syndrome (1)  Sickle cell anaemia (1) | **Cryopreservation** 26.2 (5.8)  **Transplantation**  33.5 (4.8) | Frozen (31)  Amount of ovary transplanted, median 16 (range 4–32)  (6 × 4 × 1.5 mm) | Laparoscopy (31) | Pelvic side wall (27), SC (3), SC+ Pelvic side wall (1) | Pregnancies  Live births  Follow up 2.2 years (range: 0.6–12.0) |
| Roux et al. 2019  (France  ) | HL (51), NHL (21), Ewing's Sarcoma (5), AML (5), ALL (3), Myeloproliferative syndrome (1), Synovial sarcoma (2), Rectal Cancer (2), Myxofibrosarcoma (1), Abdominal myoma (1), Spinal ependymoma (1), Neuroblastoma (1), Mesothelium (1), Borderline ovarian, Cancer (4), Breast, Cancer (5), Cervical Ca (1), Sickle cell disease (2), Thalassemia (2), Fanconi Anaemia (1), Periarteritis nodosa (1), Systemic mastocytosis (1), Schwachman-Diamond syndrome (1), Neurolupus (1) | **Cryopreservation**  26.3 (5.8)  **Transplantation**  32.8 (4.6) | Frozen (114)  Amount and size of tissue not given. | 2 step laparoscopy (69)  Not described (45) | Remaining ovary (105) | Return of ovarian activity  Pregnancies  Live births  6 months follow up |
| Sanchez et al. 2007  (Spain) | Hysterectomy + BSO (12) | **Transplantation**  40.5 (2.9) | Fresh (12) | Laparoscopy (12) | Contralateral ovary (12) | AMH and FSH at baseline and post transplantation  Follow up 2 years |
| ***Silber 2010****; Silber 2008  (USA) | POI (9) | **Transplantation**  32.5 (8) | Fresh (9)  (Donor tissue)  Amount of tissue transplanted and strip size not given | Mini laparotomy (9) | Recipient ovary (8),  Whole ovary transplant (1) | FSH, LH and oestrogen at baseline and post transplantation  Return of menstrual activity  Pregnancies  Live births |
| Silber et al. 2018  (USA) | Brain tumour (1)  HL (4)  Leukaemia (3)  MS (1)  POI (3)  Synovial cancer (1) | **Cryopreservation** 23.8 (4)  **Transplantation**  31.2 (3.9) | Frozen (12)  Frozen donor (1)  25% of thawed tissue transplanted  (10x10x1.5mm) | Mini laparotomy (13) | Remaining ovary (13) | FSH and AMH at baseline and post-transplant  Oestrogen at baseline and post-transplant  Return of menstruation  Pregnancies  Live births  Follow up >5 years |
| Suzuki et al. 2015  (Japan) | POI (37) | **Cryopreservation**  37 (4.7)  **Transplantation**  37 (4.7)^5^ | Frozen (37)  An average of 60 ovarian cubes transplanted (1.5mm^3^) | Laparoscopy (37) | Serosa of fallopian tubes n (37) | Pregnancies  Live births |
| Suzuki et al. 2019  (Japan) |  | **Cryopreservation** 35.5 (3.8)  **Transplantation**  40.6 (3.7) | Frozen (8)  11 fragments transplanted (mean)  Size of fragments not given |  | Remaining ovary + pelvic side wall (6), Remaining ovary (2) | AMH at baseline and post-transplant  Pregnancies  Ongoing pregnancies |
| Van der ven et al. 2016^2^  (Germany, Austria and Switzerland) | Breast cancer (14)  HL (10)  Other (14)  Benign disease (2) | **Cryopreservation** 31 (5.9)  **Transplantation**  35 (5.2) | Frozen (40)  15-20% of ovaries transplanted  Size not given | Laparotomy (2)  Laparoscopy (38) | Remaining ovary (4), Peritoneal pocket (30), Remaining ovary + peritoneal pocket (6) | Return of menstrual activity  Pregnancies  Live births  Follow up > 1 year |
| Zhai et al. 2016  (Japan, China and US) | POI (14) | **Transplantation**  29.2 (4.2) | Fresh (14) | Laparoscopy (14) | Serosa of fallopian tube (14) | Pregnancies  Live births  Follow up 1 year |
| **Case reports/case series**  **(Studies with < 5 patients)** | | | | | | |
| Akar et al. 2011  (USA) | HL | **Cryopreservation**  32  **Transplantation**  38 | Frozen (1)  11 strips | Robotic assisted laparoscopy | Remaining ovary + pelvic side wall | FSH, LH and oestrogen at baseline and post-transplant  Return of menstruation  6 month follow up |
| Almodin et al. 2015  (Brazil) | POI | Transplantation  31 | Fresh  4 fragments  (2x5x1.5mm) | Laparotomy | Recipient ovary | FSH, LH and oestrogen at baseline and post-transplant  Return of menstruation  18 months follow up |
| Azem et al. 2012  (Israel) | HL | **Cryopreservation**  28  **Transplantation**  38 | Frozen | Mini laparotomy | Pelvic side wall | FSH and LH at baseline and post-transplant  Oestrogen post-transplant  Pregnancy  Live birth |
| Burmeister et al. 2013  (Australia) | Breast Cancer (1)  Not specified (1) | **Cryopreservation**  38(1.4)  **Transplantation**  45 (3.8) | Frozen (2)  10 fragments (5x5x1mm) | Mini laparotomy | Remaining ovaries | Oestrogen at base line and post-transplant  FSH post-transplant  Return of menstruation  Pregnancy  Live birth |
| Bystrova 2019  (Russia) | HL (1) | **Cryopreservation** 26  **Transplantation** 28 | Frozen  2 fragments (5x5 mm) | Mini laparotomy | Remaining ovary | FSH, LH and AMH at baseline and post-transplant  Return of menstruation  Pregnancy  Live birth |
| Dunlop et al. 2016  (UK) | Wilm’s tumour | **Cryopreservation**  22  **Transplantation**  32 | Frozen  8 fragments  (10x10x5mm) | Laparoscopy | Remaining ovary | FSH, LH, AMH and oestrogen post-transplant  Pregnancy  Live birth |
| Fabbri et al 2018  (Italy) | Cystic teratoma | **Cryopreservation**  24  **Transplantation**  34 | Frozen  15 strips  (10x2mm) | Laparoscopy + SC | Remaining ovary + pelvic side wall + SC abdominal | FSH, LH and oestrogen post-transplant |
| Fabbri et al. 2014  (Italy) | Colorectal Cancer (1)  HL (1)  Breast Cancer (1) | **Cryopreservation**  28.6 (5.5)  **Transplantation**  36.5 (5.7) | Frozen  Average of  22 strips (size not given). | Laparoscopy (2)  SD (1) | Remaining ovary + pelvic side wall (2),  SD abdominal wall (1) | FSH and LH at baseline and post-transplant  Oestrogen post-transplant  Return of menstruation |
| Fàbregues et al. 2017  (Spain) | Breast cancer (3)  Nasopharyngeal cancer (1) | **Cryopreservation**  35.3 (2.8)  **Transplantation**  40.3 (3.6) | Frozen  Average of 10 pieces transplanted. Size not given. | Laparoscopy (4) | Remaining ovary | FSH, LH and oestrogen at baseline only.  Return of menstruation  Live birth |
| Fajau-Prevot et al. 2017  (France) | Ewing’s Sarcoma | **Cryopreservation**  32  **Transplantation**  37 | Frozen  21 strips  (1.5mm) | 2 step Laparoscopy | Remaining ovary + Pelvic side wall | AMH, FSH and LH at baseline and post-transplant.  Return of menstruation  Pregnancy  Live birth |
| Haixia et al. 2019  (China) | POI | **Cryopreservation**  28  **Transplantation**  29 | Frozen  Amount not given  (1x1x1mm) | Laparoscopy | Pelvic side wall | Pregnancy  Live birth |
| Hilders et al. 2004  (The Netherlands) | Cervical cancer | **Transplantation**  29 | Fresh | SC | SC (arm) | Not given |
| Isachenko et al. 2012  (Germany) | HL | **Cryopreservation**  25  **Transplantation**  32 | Frozen  Max 8 strips  2.5x1x1mm | Laparoscopy | Pelvic side wall | Return of menstruation  Pregnancy  Live birth |
| Isachenko et al. 2013  (Germany) | Ewing’s Sarcoma | **Cryopreservation**  25  **Transplantation**  30 | Frozen  9 pieces  5x3x1mm | Laparoscopy | Pelvic side wall | FSH and oestrogen at baseline and post-transplant Return of menstruation  Pregnancies  Live births |
| ***Kim et al. 2012***  (Kim et al 2004; Kim et al. 2009)  (South Korea) | Cervical Cancer (3)  Breast Cancer (1)  HL (1) | **Cryopreservation**  31 (4.1  **Transplantation**  35.8 (4) | Frozen  8-20 pieces  5x5x1mm | IM | Between rectus muscle and rectus sheath | FSH at baseline and post-transplant  10 year follow up |
| Kiran et al. 2004  (Turkey) | TAH + BSO | **Transplantation**  44 | Fresh  10 strips  4x20x2mm | IM | Rectus muscle | FSH post-transplant  6 months follow up |
| Kiseleva et al. 2015  (Russia) | Thyroid cancer | **Cryopreservation**  24  **Transplantation**  26 | Frozen  Amount and size of tissue not given | Laparoscopy | Remaining ovary | FSH, LH and AMH at baseline and post-transplant  Pregnancy  Live birth |
| Kodama et al. 2010  (Japan) | Caesarean hysterectomy | **Transplantation**  34 | Fresh | Laparotomy | Pelvic sidewall | FSH, LH and oestrogen post-transplant  4.5 year follow up |
| Kristensen et al. 2017  (Denmark) | Ovarian Cancer | **Cryopreservation**  23  **Transplantation**  32 | Frozen  18 pieces |  | Laparoscopic assisted mini laparotomy | FSH, LH and oestrogen post-transplant  Menstruation  Pregnancy  Live birth |
| Laufer et al. 2010  (USA) | Wilms’ tumour (3) | **Transplantation)**  3 (1.8) | Fresh  Amount of tissue transplanted, and strip size not given | IM | Deltoid muscle (axilla) | Menstruation (menarche)  10 year follow up |
| Lee at al. 2018  (Korea) | Rectal Cancer | **Cryopreservation**  30  **Transplantation**  34 | Frozen  Amount not described  Size 5x5x1mm | Laparoscopy | Pelvic side wall | FSH, LH, AMH and oestrogen at baseline and post-transplant  Pregnancy |
| Lorenzo et al. 2016  (Argentina) | Askin Tumour | **Cryopreservation**  28  **Transplantation**  32 | Frozen  12 strips  2mm | Laparoscopy | Remaining ovary | Menstruation  Pregnancy  Live birth |
| Mardesic et al. 2017  (Prague) | Breast Cancer | **Cryopreservation**  29  **Transplantation**  36 | Frozen  Amount of tissue transplanted, and size not given | Laparoscopy | Remaining ovary | FSH, LH, AMH and oestrogen at baseline and post-transplant  Menstruation  Pregnancy |
| Mathews et al. 2018  (UK and Denmark) | Beta Thalassemia | **Cryopreservation**  9  **Transplantation**  21 | Frozen  7 fragments, 1-2mm | Laparoscopy | Remaining ovary + pelvic side wall | FSH, LH, AMH and oestrogen at baseline and post-transplant  Menstruation  Pregnancy  Live birth |
| Mhatre et al. 2006 | Turner’s syndrome (2)  POI (1) | **Donor age** 26,38 and 21  **Recipient age**  17,15,22 | Fresh (donor)  Various amount of tissue  Size nor given | Laparotomy | Host ovary | 9 months to 3 years follow up |
| Milenkovic et al. 2017  (Sweden) | HL | **Cryopreservation**  27  **Transplantation**  31 | Frozen  7 strips  Size not given | Mini laparotomy | Remaining ovary + pelvic side wall | FSH, LH, AMH and oestrogen at baseline and post-transplant  Menstruation  Pregnancy  Live birth |
| Muller et al. 2012  (Germany) | HL | **Cryopreservation**  25  **Transplantation**  30 | Frozen  Amount of tissue transplanted, and size not given | Laparoscopy | Pelvic side wall | FSH, LH, AMH and post-transplant  Menstruation  Pregnancy  Live birth |
| Ozkavakcu et al. 2019  (Turkey) | ALL | **Cryopreservation**  20  **Transplantation**  27 | Frozen  9 strips  0.5x1x1cm | Laparoscopy | Pelvic side wall | FSH at baseline and post-transplant  Pregnancy  Live birth |
| Povoa et al. 2016  (Portugal) | Adnexectomy (secondary to a mass) + congenital absence of right ovary | **Cryopreservation**  18  **Transplantation**  28 | Frozen  8 fragments  2x2mm | Laparoscopy | Pelvic side wall | FSH and oestrogen at baseline and post-transplant  Menstruation |
| Radford et al. 2001  (UK) | HL | **Cryopreservation**  36  **Transplantation**  37 | Frozen  2 strips  1x5cm | Laparoscopy | Remaining ovary + pelvic side wall | FSH, LH and oestrogen at baseline and post-transplant  Menstruation |
| Radwan et al. 2016  (Poland) | Cervical cancer | **Cryopreservation**  28  **Transplantation**  29 | Frozen  2 fragments  5x5x1mm | Laparoscopy | Pelvic side wall | FSH, LH, AMH and oestrogen at baseline and post-transplant  Menstruation |
| Revel et al. 2011  (Israel) | Beta Thalassemia | **Cryopreservation**  19  **Transplantation**  23 | Frozen  36 fragments  1.5x3x1.5mm | Laparotomy | Remaining ovary + pelvic side wall | Pregnancy  Live birth |
| ***Revelli et al. 2013****  (Biasin et al. 2015)  (Italy) | Beta Thalassemia | **Cryopreservation**  21  **Transplantation**  29 | Frozen  17 fragments  5x5x1mm | 2 step laparoscopy | Remaining ovary | FSH, LH and oestrogen at baseline and post-transplant  Menstruation  Pregnancy  Live birth |
| Rodriguez- Walberg et al. 2014  (Sweden) | Ewing’s Sarcoma | **Cryopreservation**  23  **Transplantation**  31 | Frozen  97 pieces  1x3x5mm | Laparoscopy | Remaining ovary | FSH, LH and oestrogen at baseline and post-transplant  Pregnancy  Live birth |
| Shapira et al. 2018  (Israel) | AML | **Cryopreservation**  19  **Transplantation**  32 | Frozen  6 strips  1-2mm^2^ | Mini- laparotomy | Remaining ovary | FSH, LH and oestrogen at baseline and post-transplant  Pregnancy  Live birth |
| Tammiste et al. 2019  (Estonia) | Breast cancer | **Cryopreservation**  28  **Transplantation**  30 | Frozen  5 pieces  2x5x2mm | Submuscular | Submuscular Pelvic wall | AMH, and at baseline and post-transplant  Menstruation  Pregnancy  Live birth |
| Tanbo et al. 2015  (Norway) | HL (1)  T-cell lymphoma (1) | **Cryopreservation**  28 (5.7)  **Transplantation**  32 (2.8) | Frozen  Amount of ovary transplanted not described  Size 5x5x1mm | Mini- laparotomy | Remaining ovary | Pregnancy  Live birth |
| Wølner-Hanssen et al. 2005  (Sweden) | Sjogren’s syndrome | **Cryopreservation**  32  **Transplantation**  Not given | Frozen  Amount of tissue transplanted not given  2x2x1mm | IM | **I**M | Not given |
| Yin et al. 2009  (Taiwan) | Accidental TAH + BSO | **Transplantation**  32 | Fresh | Laparotomy | Pfannenstiel  incision site | FSH, LH and oestrogen post-transplant  51 months follow up |

*Note:*

*Studies originating from the same group of authors were cross checked for references and duplication of patients identified. Some studies may therefore have less patients than originally reported (due to removal of duplications and reporting in other studies).*

* = Primary study. ^1^ Primary study used to report outcomes whilst others used to gather data in individual patients where possible. ^2^ Data provided by Dittrich et al. not included in this study (as IPD provided). ^3^ Age at cryopreservation n= 1. ^4^ All studies published by the author in primary study combined. ^5^ Transplantation a few days after IVA, freezing and thawing. ^6^ IPD data provided by Professor R Dittrich. *SC*, Subcutaneous; *IM*, intramuscular; *SD*, Subdermal; *CML*, Chronic Myeloid Leukaemia; *HL*, Hodgkin lymphoma; *NHL*, Non-Hodgkin lymphoma; *AML*, Acute Myeloid Leukaemia; *PNH*, Paroxysmal Nocturnal Haemoglobinuria; *TAH + BSO*, Total abdominal hysterectomy + bilateral salpingo-oophorectomy; *MPA*, Microscopic polyangiitis; *IVA*, In vitro activation; *ALL,* Acute lymphocytic leukaemia
