# Supplementary material for: Fresh and cryopreserved ovarian tissue transplantation for preserving reproductive and endocrine function: a systematic review and individual patient data meta-analysis
Source: Hum Reprod Update. 2022 Feb 24;28(3):400–16. doi: 10.1093/humupd/dmac003 (PMC9733829; doi:10.1093/humupd/dmac003)
Supplement: dmac003_Supplementary_Data [file dmac003_supplementary_data.zip › dmac003-suppl_data/Supplementary Table SI final.docx]

**Supplementary Table SI** Search strategy for Database: Ovid MEDLINE(R).

--------------------------------------------------------------------------------

1 Humans/

2 Female/

3 exp ADULT/

4 exp Middle Aged/

5 3 or 4

6 (human or female* or woman or women or adult* or middle aged).mp. [mp=title, abstract, original title, name of substance word, subject heading word, keyword heading word, protocol supplementary concept word, rare disease supplementary concept word, unique identifier, synonyms]

7 1 or 2 or 5 or 6

8 1 and 2 and 5

9 (human* and (female* or woman or women) and (adult* or middle aged)).mp. [mp=title, abstract, original title, name of substance word, subject heading word, keyword heading word, protocol supplementary concept word, rare disease supplementary concept word, unique identifier, synonyms]

10 8 or 9 (

11 exp Tissue Donors/ or tissue donor*.mp.

12 tissue transplants.mp. or exp Transplants/

13 tissue transplantation.mp. or exp Tissue Transplantation/

14 exp Transplantation, Autologous/

15 exp AUTOGRAFTS/ or autograft*.mp.

16 exp Transplantation, Heterotopic/ or heterotopic transplant.mp.

17 exp Allografts/

18 exp Heterografts/

19 (allograft* or autograft* or xenograft* or orthotopic or heterotopic or autotransplant or autotransplantation or transplant*).mp. [mp=title, abstract, original title, name of substance word, subject heading word, keyword heading word, protocol supplementary concept word, rare disease supplementary concept word, unique identifier, synonyms]

20 11 or 12 or 13 or 14 or 15 or 16 or 17 or 18 or 19

21 exp OVARY/ or ovary.mp.

22 (ovarian or ovaries).mp.

23 21 or 22

24 10 and 20 and 23
