# Supplementary material for: Fresh and cryopreserved ovarian tissue transplantation for preserving reproductive and endocrine function: a systematic review and individual patient data meta-analysis
Source: Hum Reprod Update. 2022 Feb 24;28(3):400–16. doi: 10.1093/humupd/dmac003 (PMC9733829; doi:10.1093/humupd/dmac003)
Supplement: dmac003_Supplementary_Data [file dmac003_supplementary_data.zip › dmac003-suppl_data/Supplementary Tables SIII - SIX final.docx]

| **FSH level post transplantation (IU/L)** | **Total number of participants, n** | **FSH in whom pregnancy was reported, IU/L, Mean (SD)** | **Women with at least one pregnancy** | **Women with at least one live birth** | **Studies** |
| --- | --- | --- | --- | --- | --- |
| ≤ 25 | 128 | 11.1 (5.4) | 64 | 44 | Andersen, et al. 2012; Anderson et al. 2008; Azem et al. 2012; Burmeister, et al. 2013 Bystrova et al. 2019; Callejo et al. 2001; Callejo et al. 2013; Demeestere et al. 2006; Dittrich 2008; Dittrich 2012; Donnez and Squifflet 2011; Donnez and Squiflet 2010; Donnez et al 2004; Donnez et al 2006; Donnez et al. 2007; Donnez et al. 2012; Dunlop et al. 2016; Ernst 2013; Fàbregues et al. 2017; Fajau-Prevot et al. 2017; Greve et al. 2012; Gook 2019; Imbert et al. 2014; Demesteere et al. 2015; Silber et al. 2018; Isachenko et al. 2013; Janse et al. 2011; Kiran et al. 2004; Kristensen et al. 2017; Mardesic et al. 2017; Mathews et al. 2018; Meirow et al. 2016; Meirow et al. 2005; Milenkovic et al. 2017; Muller et al. 2012; Oktay et al. 2003; Oktay et al. 2016; Ozkavakcu et al. 2019; Povoa et al. 2016; Radwan et al. 2016; Rodrigeuz- Walberg et al. 2014; Rosendahl et al. 2006; Schmidt et al. 2011; Silber et al. 2005; Stern et al. 2013; Tammiste et al. 2019; Yin et al. 2009; Beckmann et al. 2017 |
| > 25 | 43 | 48.5 (29) | 11 | 8 | Almodin et al. 2015; Andersen et al. 2012; Anderson et al. 2008; Bystrova et al. 2019; Callejo et al. 2001; Donnez et al. 2011; Donnez et al. 2008; Fabbri et al. 2018; Fabbri et al. 2014; Greve et al. 2012; Gook 2019; Imbert et al. 2014; Silber et al. 2018; Janse 2011; Kim et al 2004; Kodama et al 2010; Lee at al 2018; Meirow et al. 2016; Radford et al. 2001; Revelli et al. 2013; Schmidt et al. 2011; Shapira et al. 2018; Schmidt et al. 2004; Beckmann et al. 2017 |

**Supplementary Table SIII** Pregnancy and live births in relation to FSH (IU/L).

**Supplementary Table SIV** Pregnancy and live births in relation to oestrogen (pmol/l).

| **Oestrogen level post transplantation**  **(pmol/l)** | **Total number of participants, n** | **Oestrogen in whom pregnancies were reported, pmol/l, Mean (SD)** | **Women with at least one pregnancy** | **Women with at least one live birth** | **Studies** |
| --- | --- | --- | --- | --- | --- |
| <200 | 21 | 121 (34.8) | 7 | 4 | Almodin et al. 2015; Bystrova et al. 2019; Ernst et al. 2012; Imbert et al. 2014; Silber et al. 2018; Meirow et al. 2016; Oktay 2016; Rosendahl 2006 |
| ≥200 | 56 | 652.3 (724.4) | 19 | 15 | Anderson et al. 2008; Burmeister et al. 2013; Bystrova et al. 2019; Callejo et al 2001; Callejo et al. 2013; Donnez et al. 2010; Donnez et al. 2004; Donnez et al. 2006; Donnez et al. 2007; Donnez et al. 2011; Donnez et al. 2008; Fabbri et al.2014; Gook 2019; Imbert et al. 2014  Silber et al. 2018; Isachenko et al. 2013; Janse et al. 2011; Mardesic et al. 2017; Meirow et al. 2016; Milenkovic et al. 2017; Povoa et al. 2016; Radford et al. 2001; Radwan et al. 2016; Revelli et al. 2013; Rodriguez- Walberg et al. 2014; Silber et al. 2005; Stern et al. 2013; Schmidt et al. 2004; Beckmann et al. 2017 |

**Supplementary Table SV** Details of mode of conception for pregnancies post ovarian transplantation.

| **Source (All cases)** | **Conception (live births)** | |
| --- | --- | --- |
|  | **Natural** | **Assisted** |
| Azem 2012 |  | 1 |
| Burmeister 2013 |  | 1 |
| Callejo 2013 |  | 1 |
| Diaz Garcia 2018 | 7 | 8 |
| Dolmans 2013 | 4 | 1 |
| Donnez 2011 | 7 | 8 |
| Donnez and Squifflet 2011 | 1 |  |
| Donnez et al 2004 | 1 |  |
| Donnez et al 2005 |  | 1 |
| Donnez et al 2011 | 1 |  |
| Donnez et al 2011 (2) | 7 | 5 |
| Dunlop 2016 | 1 |  |
| Ernst 2013 | 1 |  |
| Fàbregues 2017 | 1 |  |
| Fajau-Prevot 2017 | 1 |  |
| Gook 2019 | 14 |  |
| Haixia 2019 | 1 |  |
| Hoekman 2019 | 4 | 2 |
| Imbert 2014 (+IPD) | 10 |  |
| IPD R Dittrich (Beckmann M.W. 2017) | 19 | 10 |
| Isachenko 2012 |  | 1 |
| Janse 2011 | 2 |  |
| Jensen 2015 | 13 | 23 |
| Kiseleva 2015 |  | 1 |
| Klijn 2019 | 1 | 1 |
| Lorenzo 2016 | 1 | 1 |
| Meirow 2016 | 6 | 10 |
| Milenkovic 2017 | 1 |  |
| Muller et al 2012 |  | 1 |
| Oktay 2011 | 4 |  |
| Oktay 2016 |  | 2 |
| Ozkavakcu 2019 | 1 |  |
| Poirot 2019 | 12 | 1 |
| Revel 2011 |  | 1 |
| Revelli 2013 | 1 |  |
| Rodrigeuz- Walberg 2014 | 1 |  |
| Rosendahl 2006 |  | 1 |
| Roux 2019 | 27 |  |
| Schmidt 2011 | 1 | 2 |
| Shapira 2018 | 1 | 1 |
| Silber 2007 | 4 |  |
| Silber 2008 | 8 |  |
| Silber 2018 | 13 |  |
| Suzuki 2015 |  | 3 |
| Suzuki 2019 | 2 |  |
| Tammiste 2019 | 1 |  |
| Tanbo 2015 | 1 |  |
| Van der ven 2016 | 9 | 2 |
| Zhai 2016 |  | 1 |
| **Total** | **190** | **90** |

| **Type of malignancy** | **Number of women** | **Pregnancies** | **Live births** |
| --- | --- | --- | --- |
| Breast Cancer | 54 | 14 | 13 (1 set of twins) |
| Cervical Cancer | 28 | 0 | 0 |
| Non-Hodgkin's lymphoma | 27 | 11 | 7 |
| Ovarian cancer | 12 | 4 | 4 |
| Sickle cell anaemia | 9 | 3 | 3 |

**Supplementary Table SVI** Number of pregnancies and live births in women with the five most common cancers (reported at participant level data).

**Supplementary Table SVII** Overview of ovarian tissue transplantation technique.

| **Surgical Approach** | **Number of patients** | **Source** |
| --- | --- | --- |
| Laparoscopy | 205 | Bystrova 2019; Janse 2011; Poirot 2019; Zhai 2016; Callejo, J., et al. (2013); Donnez et al 2011 (2); Donnez et al 2012; Gook 2019; Beckmann M.W. 2017 + IPD; Oktay 2004; Oktay 2016; Diaz; Garcia et al. 2018; Dunlop et al. 2016; Fabbri et al 2018; Fabbri et al. (2014); Fàbregues et al. (2017); Fajau-Prevot et al 2017; Haixia 2019; Isachenko et al. 2012; Isachenko et al. 2013; Kiseleva et al 2015 ;Lee at al 2018; Lorenzo 2016; Mardesic 2017; Mathews et al 2018; Muller et al 2012; Ozkavakcu 2019; Povoa et al 2016; Radford 2001; Radwan 2016  Revelli 2013 |
| Intramuscular (IM) | 6 | Oktay 2003; Kim et al 2012; Hilders et al 2004; Laufer 2010; Tammiste 2019 |
| Laparotomy/ Mini laparotomy | 95 | Almodin et al (2015);  Andersen, C. Y., et al. (2012);  Donnez et. Al 2008;  Meirow 2005;  Revel 2011;  Tryde Schmidt 2004; Sanchez et al. 2007; Diaz Garcia et al. 2018; Mhatre et al. 2003; Kim et al. 2012; Azem et al 2012;  Burmeister, L., et al. (2013); Donnez et al 2011; Donnez et al 2011 (2); Donnez and Squiflet 2010;Ernst 2012; Meirow (2016); Milenkovic 2017; Shapira 2018; Silber 2008; Tanbo 2015 Anderson 2008; Kristensen 2017; Rosendahl 2006;  Greve et al.(2012) |
| Subcutaneous | 12 | Oktay 2011; Bystrova 2019; Kiran 2004 |
| Submuscular | 1 | Tammiste et al. 2019 |

**Supplementary Table SVIII** Reproductive and endocrine outcomes based on site of transplant.

| **Site of transplant** | **No. of participants in whom transplant site reported** | **Endocrine function** | | **Reproductive function** | | **Studies** |
| --- | --- | --- | --- | --- | --- | --- |
|  |  | **Oestrogen n, pre transplant, post-transplant Mean (SD) (pmol/l)** | **FSH n, pre transplant, post-transplant Mean (SD) (IU/L)** | **Pregnancies** | **Live births** |  |
| Remaining ovary | 175 | 33,  104.9(143.7),  387.5(419.6) | 55,  71.5(44.6),  25.3(28.5) | 52 | 34 | Greve et al. 2012; Janse et al. 2011; Meirow et al. 2016; Schmidt et al. 2005; Schmidt et al. 2011; Sanchez et al 2007; Andersen et al. 2012; Andersen et al. 2008; Donnez and Squifflet 2011; Donnez et al. 2005; Donnez et al. 2007; Donnez et al. 2011; Suzuki 2019; Oktay 2016; Almodin et al. 2015; Burmeister et al. 2013; Ernst et al. 2012;  Fàbregues et al. 2017; Lorenzo 2016; Mardesic 2017; Revelli 2013;  Diaz-Garcia 2018; Rodrigeuz- Walberg et al. 2014;  Shapira et al. 2018;  Tanbo et al. 2015;  Schmidt et al. 2004; Roux et al. 2019 |
| Pelvic side wall or peritoneum | 184 | 22,  207.5(245.1),  1204.4(1164.3) | 25,  58.8(38.5)  14.4 (13.2) | 41 | 37 | Greve et al. 2012; Poirot et al. 2019; Schmidt et al. 2011; Zhai et al. 2016; Callejo et al. 2013; Donnez et al. 2004, Donnez et al. 2005; Donnez et al. 2011; Donnez et al. 2012; Gook et al. 2019; Bekcman et al. 2017; Azem et al. 2012; Isachenko et al 2012; Kristensen 2017; Lee et al. 2018; Muller et al. 2012; Ozkavakcu et al. 2019; Povoa et al. 2016; Radwan et al. 2016; Kodama et al. 2010; Diaz-Garcia 2018, Roux 2019 |
| Pelvic side wall/peritoneum + remaining ovary | 50 | 14,  172(144),  1922(3257) | 24,  84.7 (47.6)  20.5 (21.1) | 28 | 10 | Greve et al. 2012; Imbert et. Al 2014  Schmidt et al. 2011; Donnez et al. 2006; Donnez et al 2011; Gook 2019; Suzuki et al. 2019; Beckmann et al. 2017; Akar et al. 2011; Ernst et al. 2013; Fabbri et al. 2014; Fajau-Prevot et al. 2017; Mathews et al. 2018; Milenkovic et al. 2017; Radford et al. 2001; Revel et al. 2011 |
| Pelvic side wall + abdominal wall + remaining ovary | 8 | - | 7,  62.9 (28.1)  10.8 (8.5) | 4 | 1 | Greve et al. 2012; Schmidt 2005; Schmidt et al. 2011; Anderson et al. 2008 |
| Intramuscular (IM) | 6 | - | - | - | - | Oktay 2003; Hilders et al 2004; Laufer 2010; Tammiste 2019 |
| Subcutaneous | 14 | - | - | 4 | 3 | Oktay 2011; Bystrova 2019; Callejo 2001; Kiran 2004 |
| Submuscular | 3 | - | - | - | 1 | Callejo 2001; Tammiste et al. 2019 |

**Supplementary Table SIX** Characteristics of studies with and without participant level data.

| **Characteristics of studies with and without participant level data** | | | | | | | | | |
| --- | --- | --- | --- | --- | --- | --- | --- | --- | --- |
| **IPD data** | Associated publication(s) | Number of participants | Age at cryopreservation  (Mean, SD) | Age at transplantation  (Mean, SD) | Cryopreservation technique | Transplantation technique | Information provided on endocrine function (Yes/No) | Pregnancy rates (%) | Live birth rates (%) |
| Australia | Gook et al. 2019 | 35 | 29.1 (6.1) | 35.5 (5.7) | Slow freezing | Laparoscopy | Yes | 29 | 11 |
| Belgium | Imbert et al. 2014; Demeestere et al. 2015) | 12 | 27.5 (5.8) | 33.1 (5.1) | Slow freezing | 2 step laparoscopy | Yes | 50 | 50 |
| Denmark | Hjorth et al. 2020, Jensen et al. 2015 | 64 | 30 (5.8) | 33.7 (5.6) | Slow freezing | Laparoscopy + mini-laparotomy | Yes | 52 | 24 |
| Germany | Beckman et al. 2017 | 86 | 30.5 (5.4) | 35.3 (4.9) | Slow freezing | Laparoscopy | Yes | 24 | 20 |
| Russia | Bystrova et al. 2019 | 10 | 30.8 (4.4) | 31.8 (4.4) | Slow freezing | Laparoscopy | Yes | n/a | n/a |
| USA | Silber et al. 2018; Silber et al. 2010, Silber et el. 200 | 13 | 23.8 (4) | 31.2 (3.9) | Slow Freezing + Vitrification | Mini-laparotomy + laparoscopy | Yes | 77 | 69 |
| Israel | Meirow et al. 2016 | 20 | 28.7 (7.5) | 34.4 (6.9) | Slow freezing | Mini-laparotomy | Yes | 50 | 30 |
| The Netherlands | Hoekman et al. 2019 | 7 | 27 (4.7) | 33.4 (5.8) | Slow freezing | Not explicitly given | No | 57 | 57 |
| **Non IPD data** | Associated publication(s) | Number of participants | Age at cryopreservation  (Mean, SD) | Age at transplantation  (Mean, SD) | Cryopreservation technique | Transplantation technique | Information provided on endocrine function (Yes/No) | Pregnancy rates (%) | Live birth rates (%) |
| Belgium | Donnez et al 2004; Donnez et al. 2005; Donnez et al. 2006; Donnez et al. 2007; Camboni et al. 2008; Donnez et al. 2008; Donnez and Squifflet 2010; Donnez et al. 2011; Donnez and Squifflet 2011) | 17 | 24 (5.1) | 29.7 (4.7) | Slow freezing | Laparoscopy | Yes | 77 | 30 |
| China | Zhai et al. 2016 | 14 | n/a (fresh transplantation) | 29.2 (4.2) | Vitrification | Laparoscopy | No | 7 | 7 |
| France | Poirot et al. 2019 | 31 | 26.2 (5.8) | 33.5 (4.8) | Slow freezing | Laparoscopy | No | 26 | 23 |
| France | Roux et al. 2019; Amiot et al. 2017 | 114 | 26.3 (5.8) | 32.8 (4.6) |  | Laparoscopy | No | 34 | 25 |
| Japan | Suzuki et al. 2015 | 37 | 37 (4.7) | 37 (4.7) | Vitrification | Laparoscopy | No | 8 | 5 |
| Japan | Suzuki et al. 2019 | 8 | 35.5 (3.8) | 40.6 (3.7) | Vitrification | Not explicitly given | Yes | 25 | Not given |
| Russia | Lisyanskya et al. 2009 (conference abstract) | 16 | Not given | Not given | Not given | Not given | Not given | Not given | Not given |
| Spain | Diaz-Garcia et al. 2018 | 44 | 34.3 (7.2) | 38.9 (4.1) | Slow freezing | Laparoscopy + mini-laparotomy | No | 27 | 18 |
| USA | Oktay et al. 2016; Oktay et el. 2003; Oktay et el 2004; Oktay et el 2011 | 6 | 27 (4.7) | 34.5 (2.4) | Slow freezing | Laparoscopy | Yes | 50 | 33 |
| USA | Kim et al. 2012 | 6 | 31 (4.1 | 35.8 (4) | Slow freezing | Between the rectus muscle and the rectus sheath | No | 4 embryos but no pregnancies reported | Not given |
